# Anthocyanin‐Biofortified Colored Wheat Prevents High Fat Diet–Induced Alterations in Mice: Nutrigenomics Studies

**DOI:** 10.1002/mnfr.201900999

**Published:** 2020-05-18

**Authors:** Saloni Sharma, Pragyanshu Khare, Ashish Kumar, Venkatesh Chunduri, Aman Kumar, Payal Kapoor, Priyanka Mangal, Kanthi Kiran Kondepudi, Mahendra Bishnoi, Monika Garg

**Affiliations:** ^1^ Agri‐Biotechnology Division National Agri‐Food Biotechnology Institute (NABI) S.A.S. Nagar Mohali Punjab 140306 India; ^2^ Food and Nutritional Biotechnology Division National Agri‐Food Biotechnology Institute (NABI) S.A.S. Nagar Mohali Punjab 140306 India; ^3^ Department of Natural Products National Institute of Pharmaceutical Education and Research (NIPER) S.A.S. Nagar Mohali Punjab 160062 India

**Keywords:** anthocyanins, black wheat, high‐fat diets, obesity, whole wheat

## Abstract

**Scope:**

Effective health‐promoting results of either anthocyanins or whole wheat against chronic diseases are well reported. The current study is designed to understand the effect and underlying mechanism of anthocyanins‐biofortified whole wheat on high‐fat diet (HF)‐induced obesity and its comorbidities.

**Method and Results:**

Mice are fed a HFD supplemented with isoenergetic white, purple, or black whole wheat for 12 weeks and analyzed by physiological, biochemical, and nutrigenomics studies (qRT‐PCR and RNA‐Seq analysis). Black wheat significantly reduces body weight gain and fat pad. Both black and purple wheats reduce total cholesterol, triglyceride, and free fatty acid levels in serum, with the restoration of blood glucose and insulin resistance. Black wheat significantly elevates the expression of enzymes related to fatty acid balancing, β‐oxidation, and oxidative stress that supported the biochemical and physiological positive outcomes. Moreover, the transcriptome analysis of adipose and liver tissue reveals activation of multiple pathways and genes related to fatty acid‐β oxidation (*crat, acca2*, *lonp2* etc.), antioxidative enzymes (*gpx1, sod1, nxnl1* etc.), along with balancing of fatty acid metabolism specifically in black wheat supplemented mice.

**Conclusion:**

Taken together, the results suggest that the incorporation of colored wheat (especially black wheat) in the diet can prevent obesity and related metabolic complications.

## Graphical abstract

The health‐promoting effect of fruit anthocyanins and whole wheat is well‐studied. This study involves anthocyanin‐rich black and purple wheat and diet‐induced obesity. Black wheat reduces body weight and fat pad gain. The lipid profile, glucose metabolism, and insulin sensitivity is restored by adipose tissue balancing through combined fat cell differentiation, fatty acid β‐oxidation, and antioxidants, leading to no ectopic fat deposition.

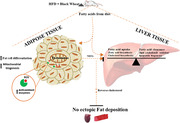

## 1 Introduction

The health benefit of whole cereals intake has been shown by numerous clinical and epidemiological studies.^[^ ^1^, ^2^, ^3^, ^4^ ^]^ Cereals are the major staple crops of the world population that provide about half of the recommended energy supply. In cereals, wheat is the most common staple cereal that can be used in various forms of foods because of its good rheological properties. Although health benefits of whole wheat were long known from its richness in dietary fiber, phytochemicals, and minerals present in the outer layers, that is, aleurone and pericarp,^[^ ^5^, ^6^, ^7^ ^]^ the existence of anthocyanins in pigmented wheat has added up, in the list of health‐promoting components. The anthocyanins belong to the flavonoid group of phytochemicals, known to possess potent antioxidant power.^[^ ^8^ ^]^ Their protective and preventive effects had been shown by the number of researchers on diet‐induced metabolic disorders.^[^ ^9^, ^10^, ^11^, ^12^ ^]^

One of the primary causal factors of obesity is the chronic intake of energy‐dense food. The obesity may cause dyslipidemia, insulin resistance, hyperglycemia, and its co‐morbidities. This chronic disorder arises because of the energy imbalance triggered by long term intake of the excess energy over expenditure leading to the storage of excess energy in the white adipose tissue (WAT).^[^ ^13^ ^]^ Once adipose tissue becomes saturated with excess energy in the form of lipid/fat, it leads to adipose tissue dysfunction (imbalance in pro‐ and anti‐inflammatory adipokines)^[^ ^14^ ^]^ and the body starts storing fat in ectopic depots like the liver and muscles, development of insulin resistance, and other linked metabolic disorders.^[^ ^15^, ^16^, ^17^, ^18^ ^]^ Thus, adipose and liver tissues are known to be the most important contributors for maintaining whole‐body metabolic homeostasis by maintaining lipid metabolism.^[^ ^19^, ^20^ ^]^ Recent studies have indicated that commonly consumed polyphenols and anthocyanins‐rich diets suppressed the co‐morbidities of high fat diet (HFD) induced metabolic disorders by enhancing the energy expenditure, fat utilization and modulating homeostasis.^[^ ^5^, ^21^, ^22^ ^]^ However, the effect of native colored wheat anthocyanins on the body, its underlying mechanism, involvement and interaction of various metabolic pathways in the energy balancing organs, that is, adipose tissue and liver is poorly understood. It is feasible by the “Nutrigenomics approach” in which transcriptome profiling is one of the first recommended step to understand the mechanistic effects of bioactive food components. With it, the effect of multiple pathways can be studied together.^[^ ^23^, ^24^, ^25^ ^]^

In our previous study,^[^ ^26^ ^]^ we have observed the advantage of anthocyanin‐rich color wheat over white wheat biochemically as well as in the in vitro cell line studies. The present study was designed to understand the effect and underlying mechanism of anthocyanins biofortified whole colored wheat on HFD induced obesity. The comparative transcriptome data of murine adipose and liver tissue depicted how differential gene expression of various metabolic pathways led to the attenuation of adverse effects of HFD induced obesity and related comorbidities.

## 2 Experimental Section

### 2.1 Animal Procurement

The experimental protocols were approved by the Institutional Animal Ethical Committee (IEAC/15/46) of National Institute of Pharmaceutical Educational and Research (NIPER), Mohali, and were following the CPCSEA (Committee for the Purpose of Control and Supervision of Experiments on Animals) guidelines on the use and care of experimental animals. Male Swiss albino mice (6–7 weeks, old 20–22 g) were procured from the central animal facility, NIPER Mohali and acclimatized for 1 week under the standard lab conditions (temperature 22 ± 2 °C; humidity 55 ± 5%; 12‐h light/dark cycle).

### 2.2 HFD Mice Model

Mice were randomly subdivided into five groups (*n* = 6–7 per group) according to body weight namely, i) Normal Pellet Diet (NPD, control); ii) High‐fat diet (HFD); iii) White wheat HFD (WHFD; iv) Purple wheat HFD (PHFD), and v) Black wheat HFD (BHFD). Groups (iii), (iv), and (v) were fed HFD, isocaloric with group (ii), but supplemented with white, purple (total anthocyanin content (TAC) ≈ 40 ppm) and black wheat (TAC ≈ 140 ppm), respectively.^[^ ^26^ ^]^ The experiment was conducted for 12 weeks and the body weight of the mice were measured on the weekly basis. Water and diet were provided ad libitum during the entire experiment.

#### 2.2.1 Dosage Information

All HFDs were formulated according to Levin et al. and were prepared in‐house.^[^ ^27^ ^]^ Each HFD group was isocaloric to each other for the total energy as well as the fraction of energy obtained from carbohydrate, protein and fat (Table S1, Supporting Information).^[^ ^6^ ^]^ However, the control group and HFD groups were not isocaloric. The control group was fed commercially available mouse NPD (Ashirwad Diet, Chandigarh) while, 100 g of HFD was prepared by mixing 36.5% of NPD (carbohydrate, protein and fat source), 31% Lard (Fat source), 25% casein (protein source) along with cellulose, vitamin/mineral mixture, Dl‐methionine, yeast extract and sodium chloride, described in Table S1, Supporting Information. On the contrary, in 100 g of WHFD, PHFD, and BHFD groups, NPD constituent was replaced by 30.7% of respective whole wheat flours (i.e., White, purple and black wheat) in such a way that total energy from carbohydrate, fat and protein remained same in wheat supplemented and non‐supplemented HFD groups. In place of cellulose, wheat bran of respective wheats was used (Table S1, Supporting Information). Total carbohydrate, protein and fat content of the wheat varieties and NPD were outsourced to National Accreditation Board for Testing and Calibration Laboratories (NABL) accredited lab, Punjab biotechnology incubator, Mohali (Table S2, Supporting Information). All HFD groups comprised of 17.42% carbohydrate, 25.60% protein, and 56% fat as a caloric source. The daily feed of individual mouse in HFD groups was approximately 3.75 g (±0.68) that led to the consumption of 45 μg of TAC by the PHFD group and 157.5 μg by BHFD group.

### 2.3 Oral Glucose Tolerance Test (OGTT)

The OGTT was performed at the 12th week of dietary manipulation on 6 h fasted mice (7:30 am to 1:30 pm). Blood glucose concentration (0 min) was measured before the oral administration of d‐glucose at a dose of 2 g kg^−1^ body weight followed by continuous measurements of blood glucose at 15, 30, 45, 60, 90, and 120 min after glucose administration. Blood was collected from the tail vein via a tail snip method and blood glucose was measured using Glucocard (Arkray Factory Inc., Shiga, Japan).

### 2.4 Serum Biochemical Analysis

After 12 weeks of dietary manipulations, blood was collected from the tail vein and allowed to coagulate at 4 °C for 20 min. The serum was separated by centrifugation at 2000 g for 15 min at 4 °C, and stored at −80 °C for further biochemical analysis. The extracted serum was used to measure free fatty acids, total cholesterol, free cholesterol, HDL, and LDL levels using corresponding commercially available calorimetric quantitation kits (Sigma‐Aldrich, USA) as per manufacturer protocols. The ELISA kits were used to measure serum insulin (Crystal Chem, USA) and leptin (Enzo Life Sciences) levels as per the manufactures instructions.

### 2.5 RNA Isolation and qRT‐PCR

Total RNA was isolated from the liver and adipose tissue of control (NPD) and HFD‐treated groups (HFD, WHFD, PHFD, and BHFD) using Trizol.

Synthesis of cDNA was performed using a Revert Aid First Strand cDNA synthesis kit (Thermo Fisher) from 2 μg of DNA‐free total RNA following the manufacturer's instructions. The quantitative real‐time PCR expression was carried out by following SYBR Green chemistry on a real‐time PCR system (ABI PRISM 7700 Fast, Applied Biosystems, USA). The expression of selected genes was normalized by using invariant housekeeping genes; *β2microglobulin* in adipose and *GAPDH* in liver tissue. The housekeeping genes were selected after screening three housekeeping genes from both the tissues.^[^ ^28^ ^]^ The relative expression was calculated by using the 2^−ΔΔCT^ method.^[^ ^28^ ^]^

### 2.6 In Vivo Antioxidant Effect Evaluation

Animals were procured and acclimatized as explained above and were randomly subdivided into five groups (six animals each) as i) control group: fed with NPD without stress and stress groups: fed with ii) NPD; iii) white wheat; iv) purple wheat, and v) black wheat. Dosage description as given in Table S3, Supporting Information and described above. These groups receiving NPD and wheat isocaloric to NPD, respectively and stress was performed as depicted below.

**Table:** 

**Days**	**1**	**2**	**3**	**4**	**5**	**6**	**7**	**8**	**9**	**10**	**11**	**12**	**13**	**14**	**15**	**16**	**17**	**18**	**19**	**20**	**21**
**Stress**	**C**	**T**	**F**	**S**	**O**	**N**	**T_1_**	**C_1_**	**O**	**N**	**F**	**S_1_**	**T_2_**	**O**	**C_2_**	**N**	**F**	**T_1_**	**S_1_**	**O**	**C_2_**

Where: C—Cold swim (8 °C, 5 min); T—Tail pinch (1 min); F—Food and water deprivation (24 h); S—Swimming at room temperature (24± 2 °C, 20 min); O—Overnight illumination; N—No stress; T1—Tail pinch (1.5 min); C1—Cold swim (10 °C, 5 min); S1—Swimming at room temperature (24 ± 2 °C, 15 min); T2—Tail pinch (2 min); C2—Cold swim (6 °C, 5 min).

After 12 weeks of treatment, mice were sacrificed and liver tissue homogenate was prepared. Homogenate was centrifuged and the upper layer was used to estimate protein by the Bradford method. Supernatant of homogenate was used to evaluate the lipid peroxidation (Malondialdehyde assay test‐MDA)^[^ ^29^ ^]^ and oxidative enzymes like superoxide dismutase^[^ ^30^ ^]^ and glutathione peroxidase.^[^ ^31^ ^]^

### 2.7 RNA‐Seq Analysis

Two replicates of RNA samples (randomly selected) per group were subjected to RNA‐sequencing (RNA‐seq) by outsourcing to Nucleome Informatics Pvt Ltd, Hyderabad.

Quality of fastq files was checked (Fast‐QC, NCBI) and subsequently aligned to the mouse reference genome (Ensembl GRCm380) using the program STAR (version 2.4.2a)^[^ ^32^ ^]^ with quantMode GeneCounts option. The STAR program was also used to generate the uniquely mapped read counts at the gene level for gene expression quantification. The differential expression analysis (DEGs) of RNA‐seq expression profiles with biological replication was performed by software package edgeR (version 3.14.0)^[^ ^33^ ^]^ using the count matrix as input. HTS Filter was used for filtering out the constant level of low counts from pairwise conditions.^[^ ^34^ ^]^ Tables of significantly differentially expressed genes with false detection rate (FDR) < 0.05 were generated for analysis.

To study the pathways affected by the treatments, pathway enrichment matrix was generated using Reactome (https://reactome.org/PathwayBrowser/#TOOL=AT and https://reactome.org/userguide/analysis) and to know the overall gene expression in all treatments simultaneously, gene count matrix was generated using Htseq. The Reactome calculates *p* values using the statistical test for over‐representation and further crosschecks its gene sets with KEGG and related databases. Further, cutoff (FDR < 0.05) was applied on the matrix and the Heatmap was generated using R plot (version Heatmap 3.0, *n* = 2). The gene count matrix was filtered by removing lower reads per kilo base per million (RPKM) values (RPKM< 2). Expression of various genes, selected from literature/gene relative to affected pathways, that is, normalized counts in all treatments with the control group were analyzed for statistical difference and at par, genes were selected. Reactome Pathway (https://reactome.org/) and MGI database (http://www.informatics.jax.org/) were used for pathway and gene functions.

### 2.8 Statistical Analysis

Graphical and statistical significance representations of data were done by using GraphPad Prism 5 represented as mean ± SEM. Unpaired Student's *t*‐test and one‐way ANOVA followed by post hoc Tukey multiple comparison tests as appropriate were performed. XLSTAT was used for principal component analysis (PCA).

## 3 Results

### 3.1 Black Wheat Balancing the Energy Storage in Adipose Tissue

**Figure 1:**
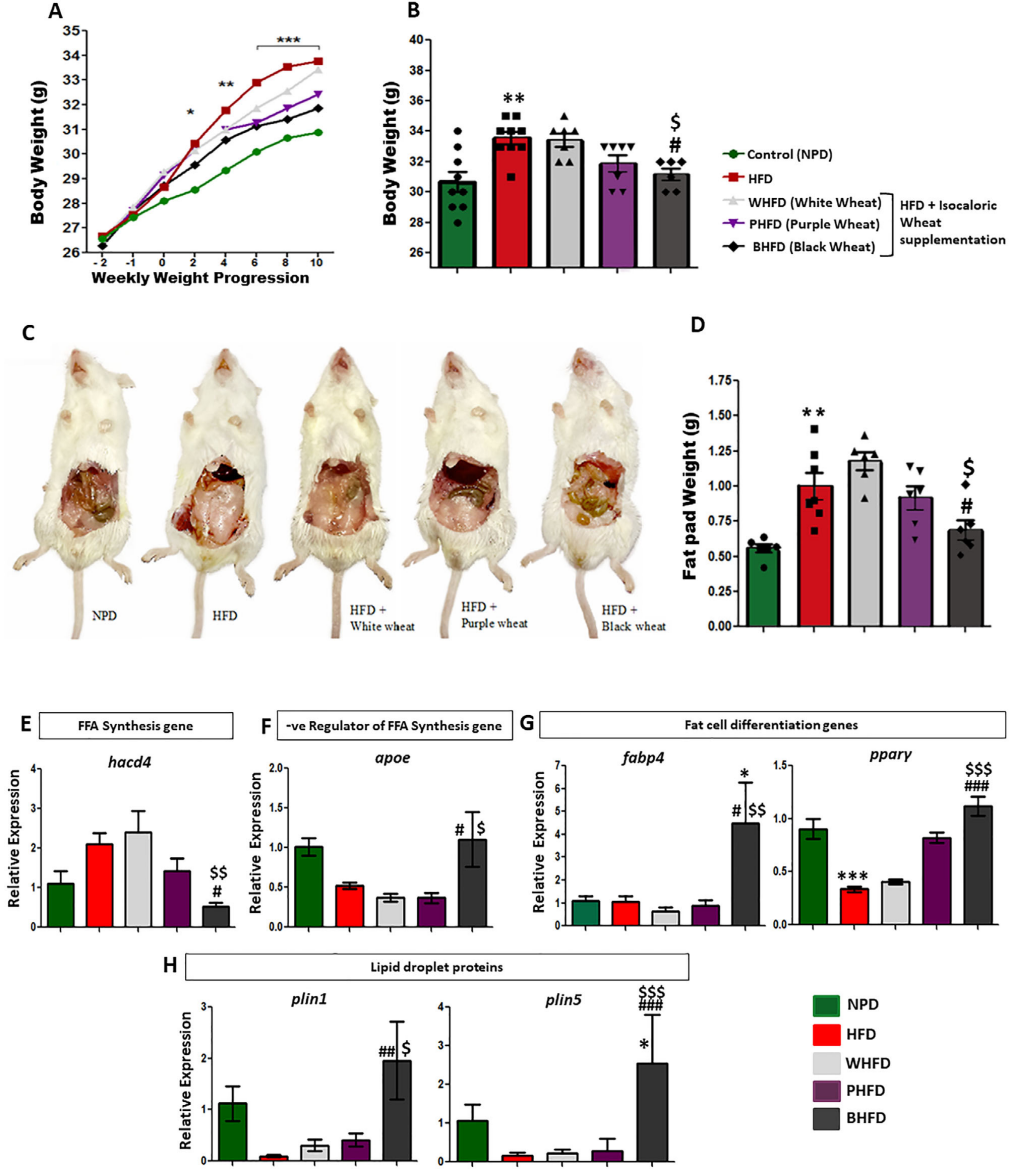
Black wheat attenuating energy imbalance created by HFD. A) Weekly progression of weight gain and total body mass in all treated groups (*n* = 7, Obesity is induced after two weeks of HFD treatment), B) total Body weight gain, C,D) fat mass depot in HFD‐treated mice (*n* = 6), E–H) real time qPCR analysis of respective genes in adipose tissue (*n* = 4–6). All values are expressed as mean ± SEM and significant level was denoted by *Pb < 0.05, **Pb < 0.05, ***Pb < 0.001, compared with the control group; #Pb < 0.05, ##Pb < 0.01, ###Pb < 0.001, compared with the HFD group; $Pb < 0.05 compared with white‐wheat‐treated group.

It is well‐established that the HFD induces obesity and its related metabolic syndrome in mice.^[^ ^9^, ^10^, ^11^, ^12^ ^]^ Based on these considerations, body weight progression (weekly) and adipose tissue weight (sacrifice day) were measured for all subjects in all replication groups. A significant increase in body weight started after two weeks in HFD group as compared to NPD (**Figure** **1**A). The body weight gain of the BHFD was significantly lower than HFD group but similar to NPD group (Figure 1B). WHFD and PHFD groups revealed no significant difference with respect to. HFD group (Figure 1A,B). The body weight gain is directly related to the excess energy storage in adipose tissue called abdominal obesity,^[^ ^13^ ^]^ which was also observed in the current study (Figure 1,D). The fat pads weight of HFD group was significantly higher than NPD. The black wheat administration significantly attenuated this increase in the fat pad, whereas, WHFD and PHFD groups showed no significant change with respect to HFD group (Figure 1C,D).

The relative expression of the fatty acid synthesis gene *hacd4* (Figure 1E) was significantly higher in HFD group as compared to the NPD group but significantly lower in BHFD group. Whereas, the relative expression of *apoe* a negative regulator of fatty acid synthesis was significantly higher in BHFD in comparison to HFD group (Figure 1F). Significant upsurge was also found in the *fabp4, pparγ, plin1*, and *plin5* in the BHFD group as compared to HFD group (Figure 1G,H). Collectively, the BHFD group showed negligible weight gain in comparison to HFD group, whereas PHFD and WHFD groups showed weight gain.

### 3.2 Color Wheat Modulating Lipid Profile and Maintaining Lipid Homeostasis

**Figure 2:**
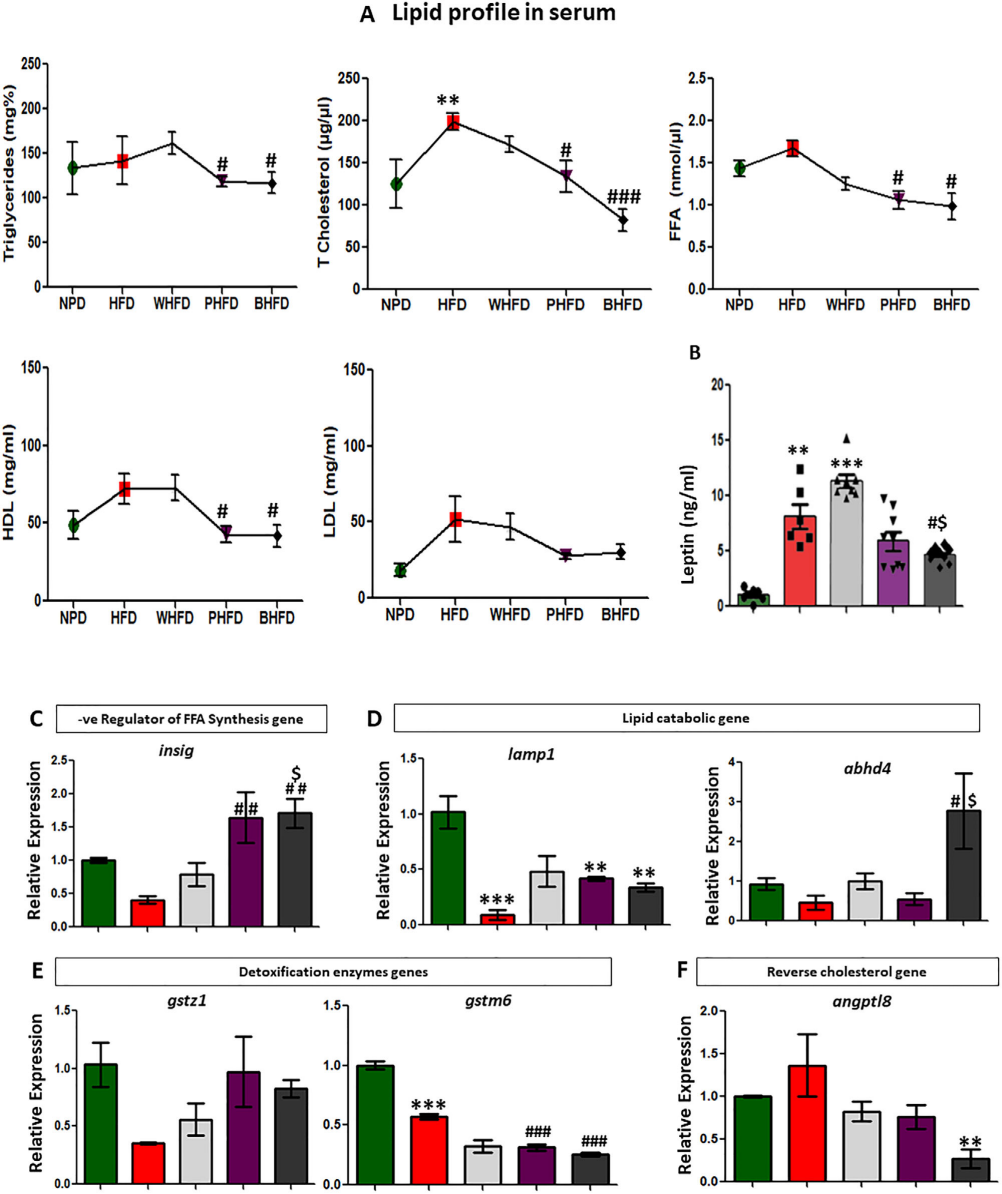
Color wheat modulating serum lipid profile and related enzymes in adipose and liver tissues. A) Lipid profile in serum (*n* = 6), B) leptin concentration in serum (*n* = 6), C–F) real time qRT‐PCR analysis of selected genes in adipose tissue (*n* = 4–6). All values are expressed as mean ± SEM and significant level was denoted by *Pb < 0.05, ***Pb < 0.001, compared with the control group; #Pb < 0.05, ##Pb < 0.01, ###Pb < 0.001, compared with the HFD group, $Pb < 0.05 compared with the white wheat treated group.

The adipose tissue is the specialized organ to store calorie‐dense, chylomicron transported triglycerides.^[^ ^35^, ^36^, ^37^, ^38^ ^]^ Once the energy storing capacity of adipose tissue develops an imbalance, the triglyceride‐rich chylomicrons and their remnants surge in the plasma, causing dyslipidemia.^[^ ^38^, ^39^ ^]^ Excessive lipids in the plasma start depositing in the liver and other organs and this ectopic fat deposition leads to lipotoxicity related diseases.^[^ ^40^ ^]^ Our results indicated that the total lipid content in the serum represented by triglycerides, total cholesterol, free fatty acid (FFA), HDL, and LDL showed significant elevation in the HFD group, compared to NPD, but upsurge was substantially reduced by the BHFD, followed by PHFD (**Figure** **2**A). The leptin level in serum was high in HFD and WHFD groups compared to the NPD, PHFD, and BHFD groups (Figure 2B). However, the phenotypes were not affected by leptin level,^[^ ^41^ ^]^ that might be because of the leptin resistance in obese mice or the leptin acts as a marker for inflammation.^[^ ^11^ ^]^

The hepatic mRNA expression indicated that *insig1*, the gene negatively regulating FA biosynthesis was upregulated in PHFD and BHFD groups as compared to the HFD group (Figure 2C). While, the lipid catabolic genes *lamp* and *abhd4* (Figure 2D) and detoxifying glutathione S‐transferase genes viz., *gstz1* and *gstm6* (Figure 2E) showed variable pattern in different wheat groups. The blood lipid regulator *angptl 8* exceptionally showed low expression in the BHFD group (Figure 2F). These results were in support of, lipid equilibrium maintenance, in the liver of all wheat‐based HFD groups.

### 3.3 Color Wheat Effecting Glucose Tolerance and Insulin Resistance

**Figure 3:**
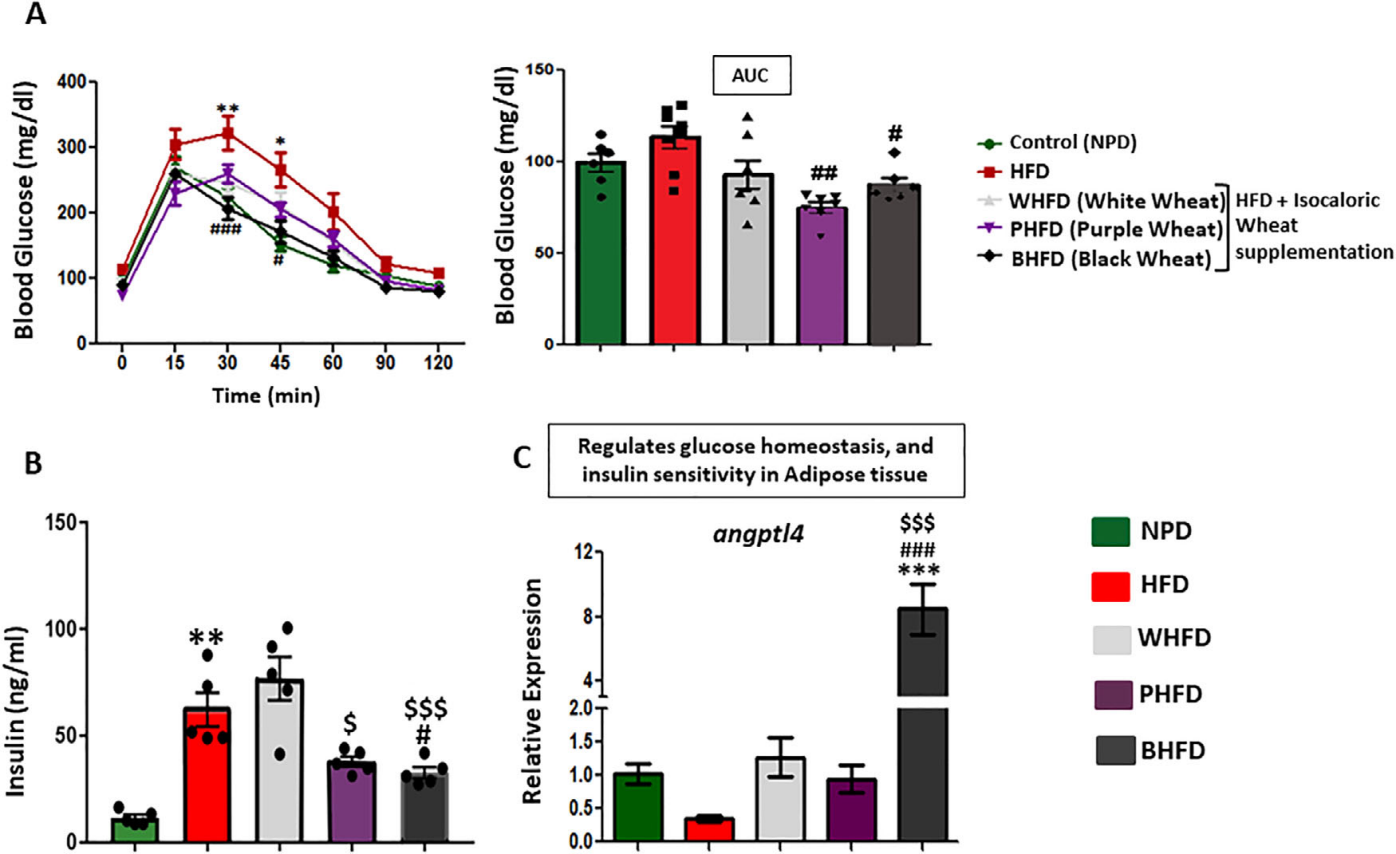
Color wheat improving the glucose/insulin homeostasis. A) Oral glucose tolerance test (OGTT) at 11 weeks (*n* = 6), B) Insulin level in serum (*n* = 5–6), C) qRT‐PCR result of *angptl4* in adipose tissue (*n* = 6). All values are expressed as mean ± SEM and significant level was denoted by *Pb < 0.05, ***Pb < 0.001, compared with the control group; #Pb < 0.05, ##Pb < 0.01, ###Pb < 0.001, compared with the HFD group; $Pb < 0.05 compared with the white wheat treated group.

Former studies had already documented a negative impact of diet‐induced obesity on insulin sensitivity leading to systemic insulin resistance.^[^ ^42^, ^43^, ^44^ ^]^ Thus to better understand the color wheat extent of action, insulin sensitivity was evaluated. After 11 weeks of HFD feeding, significant glucose intolerance was observed in the HFD group, as evidenced by, an impaired ability to lower their blood glucose level. However, BHFD and PHFD group markedly restored the glucose tolerance (**Figure** **3**A). Fed state plasma insulin level was observed to be lower in BHFD and PHFD groups as compared to HFD (Figure 3B). Even the mRNA expression of *angptl 4*, which function as a serum hormone and regulates glucose homeostasis, lipid metabolism and insulin sensitivity was significantly upsurged in adipose tissue of BHFD group (Figure 3C) than other groups. Concluding, both black and purple wheat HFD diets restored the glucose and insulin sensitivity.

### 3.4 Black Wheat Effecting Oxidative Stress Parameters

**Figure 4:**
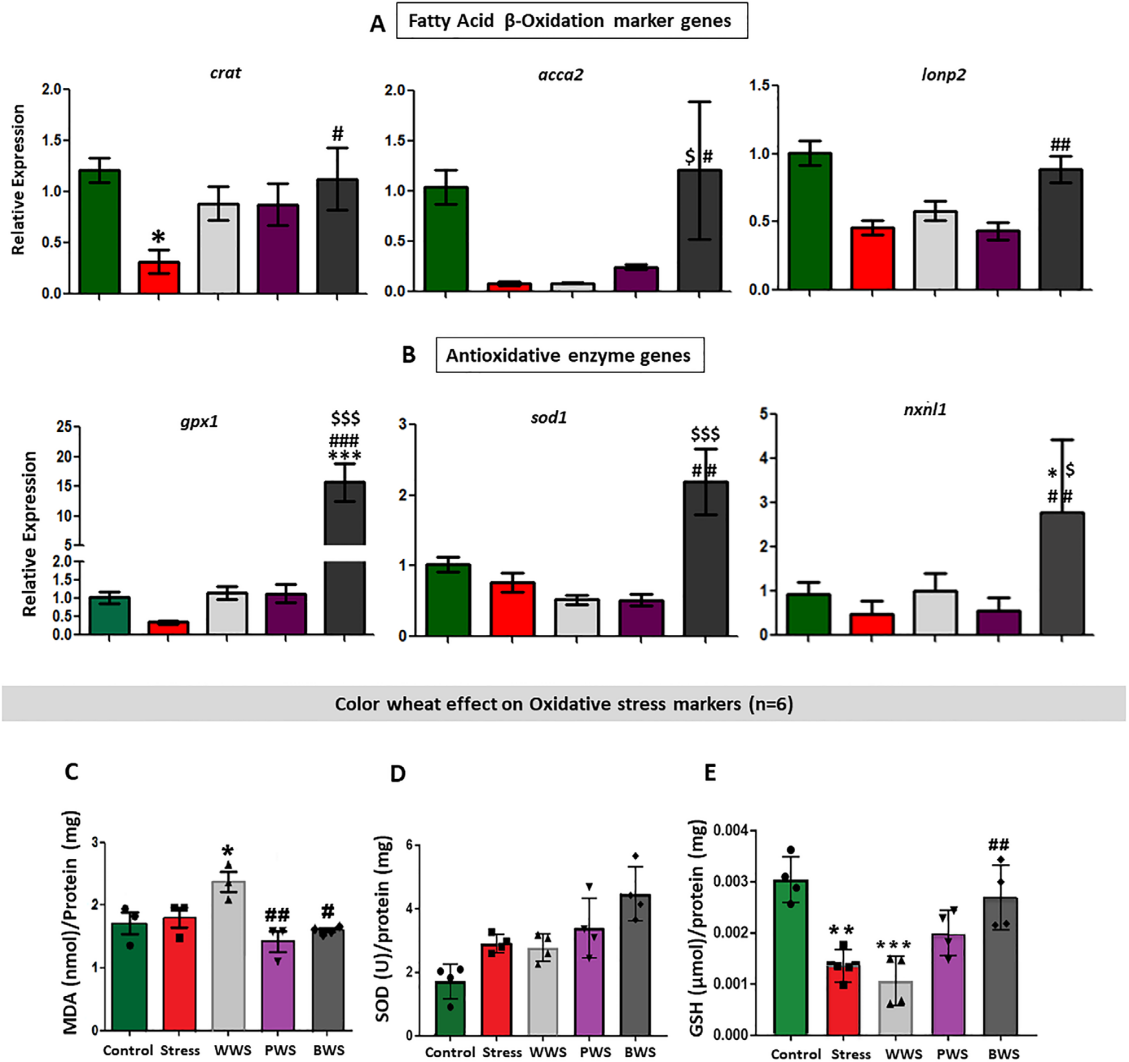
Black wheat effectively enhancing FA oxidation and decreasing ROS in adipose tissue. A,B) qRT‐PCR results revealing significant increase in fatty acid β‐oxidation marker genes and antioxidative genes in BHFD group (*n* = 6). C) Color wheat effect on oxidative stress (*n* = 6) mice model showing: Lipid peroxidation test (MDA: Malondialdehyde assay test) and antioxidative enzymes evaluation (sodium dismutase and glutathione peroxidase), respectively. All values are expressed as mean ± SEM and significant level was denoted by *Pb < 0.05, compared with the control group; #Pb < 0.05, ##Pb < 0.01, ###Pb < 0.001, compared with the HFD group; $Pb < 0.05 compared with the white‐wheat‐treated group.

The fatty acid β‐oxidation marker genes viz., *crat, acca2*, and *lonp2* were significantly up‐regulated in BHFD compared to HFD group (**Figure** **4**A). Undoubtedly, burning of fat generates energy by fatty acid oxidation and produces reactive oxygen species (ROS), leading to oxidative stress and inflammation.^[^ ^45^, ^46^ ^]^ Reduction of ROS by antioxidative enzymes, is the way to combat its harmful effect.^[^ ^47^ ^]^ In our experiment, we observed significantly up‐regulated expression of the genes coding for antioxidative enzymes, that is, *gpx1, sod1*, and *nxnl1* was significantly up‐regulated in the BHFD group compared to the other groups (Figure 4B).

Antioxidative effect of color wheat, was further visualized in vivo by evaluating stressed mouse model. Again, colored wheat specifically black wheat were definitely effective against oxidative stress markers like MDA, SOD, and GSH; their levels were lower in other groups (Figure 4C).

Thus, overall we can say that the black wheat effectively enhanced the FA oxidation and also reduced the ROS by acting as a strong in vivo antioxidant itself or by inducing antioxidant enzymes.

### 3.5 RNA Seq‐Analysis

**Figure 5:**
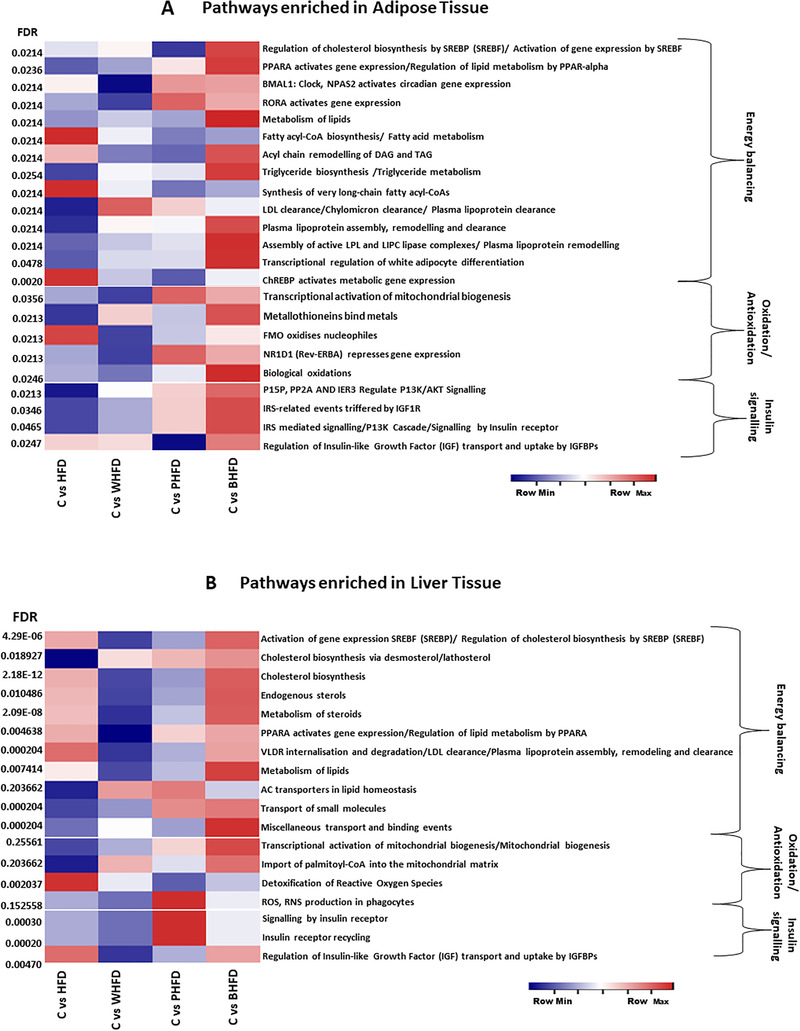
Pathways enrichment using RNA‐seq analysis. A,B) Heat map depicting differential expression of various metabolic pathways related to energy balancing, antioxidation/oxidation, and insulin signaling in adipose and liver tissue, respectively (FDR < 0.05, each Pathway description is given in Table S4, Supporting Information), C versus HFD‐expression in HFD comparative to control group, C versus WHFD‐ expression in WHFD comparative to control group, C versus PHFD‐ expression in PHFD comparative to control group, C versus BHFD‐ expression in BHFD comparative to control group.

RNA‐seq data analysis from the adipose and liver tissues, revealed a diverged gene expression pattern in different treatment groups (Figure S1, Supporting Information). In the adipose tissue, out of 1768 total affected genes, 1154 genes were up‐regulated, in the HFD group in comparison to NPD (Table S3, Supporting Information). For visualizing variable gene expression, among all treatments, pathway and gene set enrichment analysis was carried out. The pathway enrichment analysis revealed that DEGs were enriched in various metabolic pathways linked to the energy balancing, oxidative stress and insulin signalling (false detection rate [FDR] < 0.05; **Figure** 5A,B), though their magnitude of manifestation varied modestly (Table S4, Supporting Information). Regulation of cholesterol biosynthesis by sterol regulatory element binding proteins SREBP (SREBF)/ activation of gene expression by SREBP; PPARA gene expression/regulation of lipid metabolism by PPAR‐α ; metabolism of lipids; triglyceride biosynthesis/triglyceride metabolism; assembly of active lipoprotein lipase (LPL) and hepatic lipase (LIPC) complexes/ plasma lipoprotein remodelling, transcriptional regulation of white adipocyte differentiation were significantly (FDR > 0.05) up‐regulated in BHFD and down‐regulated in HFD compared to control group. Whereas, opposite has been observed for fatty acyl‐CoA biosynthesis/fatty acid metabolism, synthesis of very long‐chain fatty acyl‐CoAs and carbohydrate‐responsive element‐binding protein (ChREBP) activated metabolic gene expression, regulating energy balance by harmonizing fatty acid metabolism in adipose tissue (Figure 5A and Table S4, Supporting Information). Even the triglyceride lipid (TRL) uptake and turnover pathways in adipose tissue were significantly upregulated in BHFD (Figure 5A; energy balancing pathways; description in Table S4, Supporting Information) group in comparison to HFD group.

Next to adipose tissue, the liver is known for lipid metabolism along with a major role in distributing lipids to other organs.^[^ ^48^ ^]^ Imbalance in lipid metabolism, that is, lipid procurement and disposal, can lead to the hepatic steatosis.^[^ ^48^, ^49^ ^]^ The pathway enrichment in the liver revealed a significant up‐regulation of pathways related to cholesterol synthesis and homeostasis in the control versus HFD and control versus BHFD groups, though the later showed more up‐regulation (Figure 5B and Table S4, Supporting Information). Pathways related to oxidative stress in liver tissue indicated that mitochondrial biogenesis pathway was upsurged in BHFD followed by PHFD group (Figure 5B and Table S4, Supporting Information).

Overall, in adipose tissue insulin sensitivity regulating pathways were significantly prompted in BHFD followed by PHFD group compared to other groups, while in liver tissue PHFD showed more stimulation than BHFD group (Figure 5A,B; Table S4, Supporting Information).

To further gain insight, we analyzed individual gene expression by using a gene count matrix. Among fatty acid synthesis genes *hmgc1*, *hacd4*, *acsl4*, and *fads2* (Figure S2A, Supporting Information and Table S5, Supporting Information) were more expressed in HFD group as compared to NPD group and were significantly lowered in BHFD group. *Acadl*, *acadvl*, and *apoe* which are negative regulators of fatty acid synthesis were significantly increased in the BHFD group in comparison to HFD group (Figure S2B, Supporting Information and Table S5, Supporting Information). Previous studies mentioned that for positive energy balance, appropriate expansion of adipose tissue is essential to store surplus energy.^[^ ^35^, ^50^ ^]^ Here we also observed increased expression of fat cell differentiation inducing genes *fabp4*, *pparγ*, *arxes1*, and *adig* (Figure S2C, Supporting Information and Table S5, Supporting Information) in BHFD group. Even the expression of lipid droplet protein (LDs) genes viz.*, plin1*, *cidec*, *bscl2*, and *agpat2* (Figure S2D, Supporting Information and Table S5, Supporting Information) controlling expandability of adipose lipid droplet storage was observed to be significantly high in BHFD group.^[^ ^33^ ^]^ Higher lipid turnover rate in BHFD was depicted by enhanced expression of lipid catabolic genes, that is, *ces1d*, *ces1f*, *lipe*, and a positive regulator of triglyceride catabolic process and lipase activity gene *abhd5* (Figure S3A, Supporting Information and Table S5, Supporting Information).

As in our study, we observed the up‐regulation of transcriptional activity of mitochondrial biogenesis pathway in adipose tissue of black wheat‐based HFD group compared to NPD and HFD groups (Figure 5A and Table S4, Supporting Information). Thus, on exploring the gene count matrix, we observed significant increase in fatty acid β‐oxidation marker genes viz., *acadl; acadvl*; *acatl*; *acat3*; *acaa2*; *acaa1b*; *acadm*; *acads*; *etfb*, *gcdh*; *hsd17b4*; *hadh*; *hadha*; *por*; *ecil*; *acbd4*; *lonp2*; *etfbkmt*; and *plin5* along with the obligate enzymes required for long‐chain mitochondrial fatty acid β‐oxidation *cpt2* and *crat* in BHFD group compared to NPD and HFD group (Figure S3B,C, Supporting Information; Table S5, Supporting Information). Burning of fat by fatty acid oxidation generates energy and also produce reactive oxygen species (ROS), consequently leading to oxidative stress and inflammation causing insulin resistance and other metabolic morbidities.^[^ ^45^, ^46^ ^]^ Reduction of ROS by antioxidative enzymes is the way to combat its harmful effect.^[^ ^47^ ^]^ Here, we observed either upsurge or similar gene expression of antioxidative enzymes in all HFD groups compared to the control group, to reduce ROS to some extent. But the expressions of enzymes like glutathione peroxidases: *gpx1*, *gpx3*, *gpx4*, *gpx7*, glutathione S‐transferases: *gstt2*, *gstz1*, *gsto1*, *mgst1*, and *ltc4s*, *nxn*, *nxnl*, *seleno, sod1*, *cat, nnat* (Figure S3D, Supporting Information and Table S5, Supporting Information) showed significant upsurge in BHFD group and thus might have repressed oxidative stress to greater extent, proving to be effective antioxidant or detoxifying natural antioxidative enzymes of body (Table S5, Supporting Information). These results also support in vivo antioxidant effect (Figure 4).

To find out, correlation among different feeding groups, for differential gene expressions in adipose tissue, principal component analysis (PCA) was carried out. First principal component (PC1) representing 50.7% of variance, segregated feeding groups based on gene signature pattern (Figure 3E right plot). BHFD and control group mice showed sharp segregation in contrast to PHFD, WHFD and HFD groups, which did not clearly segregate according to significantly variable gene signature pattern along PCA1 and PCA2. It was clear from variable plot analysis, (Figure S3E, Supporting Information left plot) that the FA biosynthesis genes expression separated the HFD group. The FA β‐oxidation markers and antioxidative enzyme genes splitted BHFD group. Thus, overall we can say that black wheat effectively enhanced the FA oxidation and also reduced the ROS by acting as a strong antioxidant itself or by inducing antioxidant enzymes. This collective increase in fat cell differentiation, LDs expression and balance in fatty acid metabolism in BHFD group led to negligible weight gain compared to other HFD group, whereas, no such observation was noticed in PHFD and WHFD groups. Black wheat also effectively reduced the total lipid content in plasma by simultaneously increasing TRL uptake and turnover in adipose tissue. Similar observation was recorded in purple wheat also, but to a limited extent.

While, in liver tissue, gene count matrix revealed that genes relevant to fatty acid biosynthesis, that is, *apo c1* and *fitm1* were significantly raised in HFD mice as compared NPD, WHFD, PHFD, and BHFD group, while *fitm 2* and *dhcr 24* were highly expressed in all wheat feeding HFD groups similar to NPD group (Figure S4A, Supporting Information and Table S6, Supporting Information). *Acadvl*, *insig1*, and *scap* the negative regulators FA biosynthesis genes were upregulated in all wheat‐based HFD groups as compared to HFD group (Figure S4B, Supporting Information and Table S6, Supporting Information). Thus, we can say that lipid deposition was less in hepatocytes of all wheat fed HFD (BHFD < PHFD < WHFD) as compared to HFD mice.

Fat clearance was boosted in the liver of BHFD, PHFD, and WHFD groups (wheat fed groups) by increased lipid catabolism (Figure S4C, Supporting Information and Table S6, Supporting Information) and fatty acid β‐oxidation markers (Figure S5A, Supporting Information and Table S6, Supporting Information). *Lipa*, *lamp*, *pccb*, *acsl1*, and *acot2* the lipid catabolic enzymes were significantly upregulated in wheat fed groups (Figure S4C, Supporting Information and Table S6, Supporting Information). Fatty acid β‐oxidation marker enzymes, that is, *acaa2*, *acadvl*, *echdca1*, and *hadha* showed significant reduction in HFD and upsurge in wheat fed groups (Figure S5A, Supporting Information and Table S6, Supporting Information). On the other hand, various classes of detoxifying enzyme, glutathione S‐transferase, showed variable pattern in wheat fed groups (Figure S5B, Supporting Information and Table S6, Supporting Information). Reduction in the hepatic lipid deposition was also supported by the enhanced lipoprotein biogenesis, that maintains homeostasis in liver by functioning as key factor in inter‐organ fuel distribution, extracellular cholesterol pool management and reverse cholesterol.^[^ ^49^, ^51^ ^]^ Similarly, we also observed increased expression of *apoo*, *apon*, *ldlrap1*, and *lrpap1* genes in wheat fed groups (FigureS5C, Supporting Information and Table S6, Supporting Information). However, *angptl 8‐*a blood lipid regulator showed low expression in BHFD group (Figure S5C, Supporting Information and Table S6, Supporting Information) that was supported by qRT‐PCR results (Figure 2F). Results were in support that, lipid equilibrium was maintained in liver of all wheat‐based HFD groups.

Our observations were also confirmed by the PCA analysis of the selected genes expression in liver tissue. 53% variance was captured in PC1 and 22% in PC2 (Figure S6A, Supporting Information). Projecting different feeding groups in the variable plot of PC1 and PC2, it was revealed that control and HFD group differed apart in comparison to WHFD, PHFD, and BHFD group, later were making a single cluster because of similar gene signature (Figure S6A, Supporting Information: right‐side plot). Thus, we can say that lipid homeostasis was maintained in the liver tissue of wheat fed HFD groups compared to HFD group.

In case of adipose tissue significant upsurge was observed in BHFD group for the mRNA expression of *angptl 4*; a serum hormone that regulates glucose homeostasis, lipid metabolism and insulin sensitivity, and *nnat*; a proteolipid; which act as a positive regulator of insulin secretion, along with *adipor2;* that maintains glucose and fat homeostasis (Figure S6B, Supporting Information, Table S6, Supporting Information). Overall these results depicted that both black and purple wheat restored the glucose and insulin sensitivity even with HFD. This observation was also in support to physiological parameters (Figure 3).

## 4 Discussion

It is well established that the diet comprising of the whole grains has protective effect against the diet‐induced metabolic disorders.^[^ ^1^, ^2^, ^3^, ^4^, ^5^, ^6^ ^]^ Among the whole grains, wheat in particular, is an important component of the daily diet throughout the world. Here, we have reported for the first time the preventive effect of colored whole wheat in HFD induced mice model. Anthocyanin‐mediated preventive/protective effect against the development of obesity and T2DM had been already reported by several research groups but those were from anthocyanins extracts.^[^ ^9^, ^10^, ^11^, ^12^ ^]^ Limited research has been done on whole seeds.^[^ ^7^ ^]^ Indeed, anthocyanins are most abundant in berries, colored grapes, sweet cherries and many other fruits and vegetables, but their feasibility to the common man of the developing and underdeveloped countries is limited. Regardless of healthy components in fruits, prevalence of sugar content might increase the risk of obesity.^[^ ^52^ ^]^ With the comparative transcriptome analysis, we have tried to understand and identify novel molecular mechanism involved during the course of colored whole wheat effects on obesity and relevant co‐morbidities.

As expected, the total body weight and fat pad weight were significantly reduced in BHFD group compared to HFD, whereas the purple and white wheat HFD groups, showed non‐significant difference in the weight gain, compared to HFD group. This observation suggests that anthocyanins work in a dose dependent manner or there might be some threshold limit of anthocyanins to act as potent attenuator of weight gain. In our experiment, black wheat (≈140 ppm) had high anthocyanin content than purple wheat (≈40 ppm) and white wheat (not detected).^[^ ^26^ ^]^ Moreover, we have reported in our previous study that signature of different anthocyanidins and their derivatives, was almost similar in black (26 different types of anthocyanins) and purple wheats (23 types).^[^ ^53^ ^]^ Therefore, total anthocyanin content might be playing the major role in the prevention of HFD induced alterations. Further, comparative RNA‐seq analysis provided the insight and showed significant upsurge in BHFD group for fatty acid‐β oxidation and antioxidation response along with induction in fat cell differentiation, LDs expression and balance in fatty acid metabolism.^[^ ^54^ ^]^ Black wheat‐based diet also indicated prominent positive impact on lipid profile of plasma by increasing lipid catabolic processes in adipose tissue; even the purple wheat fed group had also shown similar effect on serum lipid profile. While RNA‐seq data of liver tissue indicated that wheat‐based HFD groups, that is, BHFD, PHFD, and WHFD maintained the lipid equilibrium.

Thus, we can propose the mechanism (for black wheat) that the anthocyanins in color wheat, work by interacting with, intracellular signaling,^[^ ^55^ ^]^ regulatory transcription factors and/or gut microbiota,^[^ ^11^ ^]^ and stimulate the adipocyte differentiation,^[^ ^13^ ^]^ expand LD proteins and increase lipid uptake to store excess energy within the adipose tissue.^[^ ^15^, ^16^, ^17^, ^49^ ^]^ Proliferation of adipocyte possibly increases mitochondrial number and surprisingly mitochondrial biogenesis also. This enhances burning of excess fat within the adipose tissue by fatty acid β‐oxidation,^[^ ^35^, ^36^ ^]^ that in turn increases the oxidative stress by generating ROS and results in the insulin resistance as later is one of the main consequences of inflammation caused by ROS.^[^ ^44^, ^46^ ^]^ The black wheat acts as very effective antioxidant and commendably detoxifies the ROS,^[^ ^43^ ^]^ and maintains glucose and insulin sensitivity.^[^ ^36^, ^44^, ^45^, ^46^, ^56^ ^]^ Lipid turnover in adipose tissue is also aided by enhanced lipid catabolic processes. Overall, these changes reduce the adipose storage and leads to reduction in body weight gain.

It was cleared that anthocyanin rich black wheat mediates the accommodation of excess energy within the fat tissue by enhancing storage capacity and fat burning, that also protects against lipotoxicity by minimizing ectopic fat deposition.^[^ ^38^ ^]^ This also leads to reduction in serum lipid profile followed by the attenuation in liver steatosis.^[^ ^11^ ^]^ Interestingly, the liver of PHFD and WHFD groups was metabolically as active as BHFD and maintained the lipid homeostasis, in spite of higher body weight gain. The obesity is one of the bases of hepatic steatosis that occurs either by the increase in FFAs and triglycerides (TGs) input or by the reduction in their output.^[^ ^41^, ^47^, ^48^ ^]^ Input of FFAs in liver depends upon their contribution from diet, adipose tissue or from de novo *lipogenesis*.^[^ ^41^, ^47^, ^48^ ^]^ Therefore, in BHFD group there would be hardly any chance to get FFAs supply to liver from adipose tissue as compared to purple and white wheat‐based groups. Even de novo *lipogenesis* contribution was seen to be nominal in all the three wheat fed groups. So extrapolating from these observations; the diet is the only contributing source of FFAs, to the liver in case of BHFD group. In PHFD and WHFD groups, diet and adipose tissue both are contributors. Moreover, their possible fate in liver depends upon lipid catabolic processes like β‐oxidation in mitochondria, synthesis of other lipids like sterols, phospholipids, and lipoprotein biogenesis that maintains the extracellular cholesterol pool and reverse cholesterol transport.^[^ ^57^ ^]^ Remarkably all these catabolic processes were observed in the all wheat‐based HFD groups to maintain the lipid equilibrium in liver. In fact, black wheat‐based group needs less input from catabolic processes as compared to purple and white wheat‐based groups. However, what other factors in purple or specifically in white wheat contribute to this type of response in liver is not clear because their performance was not at par in adipose tissue.

One possible explanation for this response might be synergetic effect of wide range of protective compounds present in the whole grain wheat. It has been proven by several research studies that whole grains have greater protective effects than the any single ingredient.^[^ ^1^, ^58^ ^]^ The whole wheat which is well acknowledged to be rich in bioactive components like polyphenols, arabinoxylans, fermentable oligosaccharides phytosterols, alkylresorcinols, etc. has been reported to reduce serum cholesterol, maintain glucose metabolism and has antioxidant properties to combat metabolic syndromes.^[^ ^1^, ^57^ ^]^ Probably all these bioactive components in whole wheat diet worked together and showed some impact on liver lipid equilibrium. To the best of our knowledge, any direct report showing the preventive effect of whole wheat diet on hepatic steatosis has not been published.

Contrarily, the purple wheat in spite of lower anthocyanin content and non‐significant effect on adipose tissue, showed positive response towards serum lipid profile and also restored the blood glucose and insulin sensitivity. Later were similar to the black wheat effect. Positive effects associated with purple wheat might be due to higher acylated anthocyanins, that are known to be more stable, bioavailable,^[^ ^25^, ^52^ ^]^ and have strong anti‐inflammatory property.^[^ ^24^ ^]^

Although, the presented study, elucidated insights into the mechanism that how anthocyanin rich whole wheat affected the expression of various genes related to energy balancing, FA β‐oxidation, antioxidation enzymes, liver lipid, and glucose homeostasis, yet less number of replicates per treatment used for transcriptome profiling is a limitation of the study. Cost is the major factor responsible for such limitation.^[^ ^22^, ^23^ ^]^ Additionally, the proposed mechanism also needs to be validate by respective protein quantification or metabolomics profiling. But in our analysis almost all RNA‐seq data positively correlated with physical and biochemical outcomes and qRT‐PCR results with appropriate and desired replicates. Moreover, the same mechanism has also been proposed for anti‐obesogenic effects of polyphenols^[^ ^59^ ^]^ except that they observed inhibiting effect on adipocyte differentiation while we have observed induction in fat‐cell differentiation. Other nutrigenomics studies reported are on different tissue.^[^ ^60^, ^61^ ^]^

The knowledge behind the preventive mechanism of whole wheat and color wheat against the metabolic disorders is still obscure, so we require deeper understanding of different bioactive components and their interaction in vivo, to elicit various interconnected pathways in diverse organs. In summary, our findings supported and proposed the insight into beneficiary/preventive mechanism of black and purple wheat in HFD induced obesity and its comorbities compared to the white whole wheat.

## Conflict of Interest

The authors declare no conflict of interest.

## Supporting information

Supporting information

Supporting information

Supporting information

Supporting information

Supporting information

Supporting information

Supporting information

Supporting information
