# Supplementary figures and images for: Anthocyanin‐Biofortified Colored Wheat Prevents High Fat Diet–Induced Alterations in Mice: Nutrigenomics Studies
Source: Mol Nutr Food Res. 2020 May 18;64(13):1900999. doi: 10.1002/mnfr.201900999 (PMC7507204; doi:10.1002/mnfr.201900999)

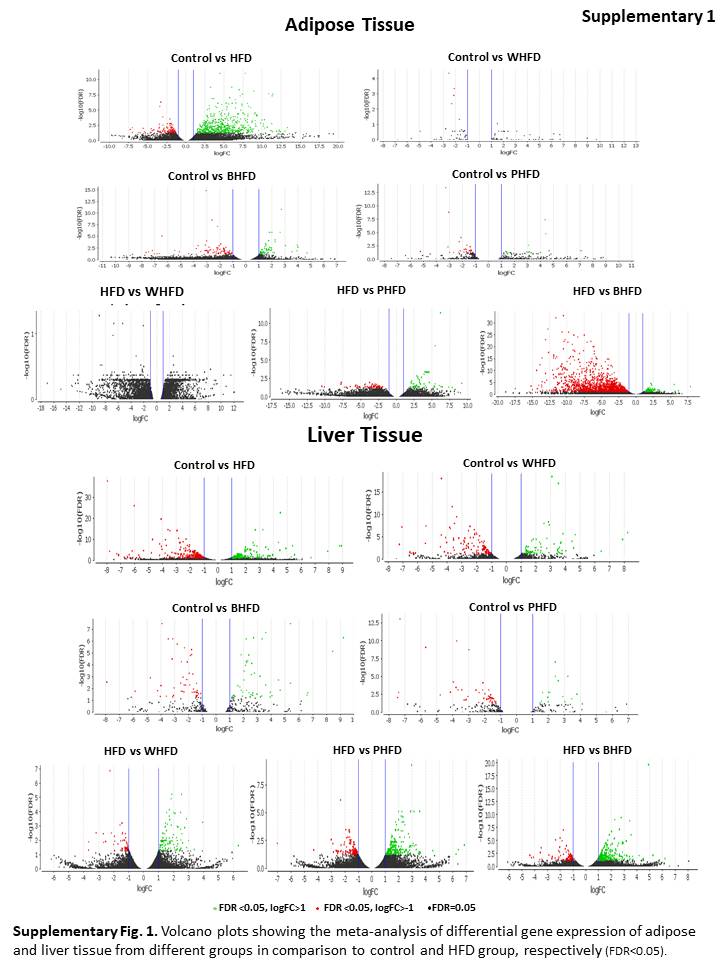

Supplement: Supplementary file 1 — Supporting information [file MNFR-64-1900999-s001.jpg]

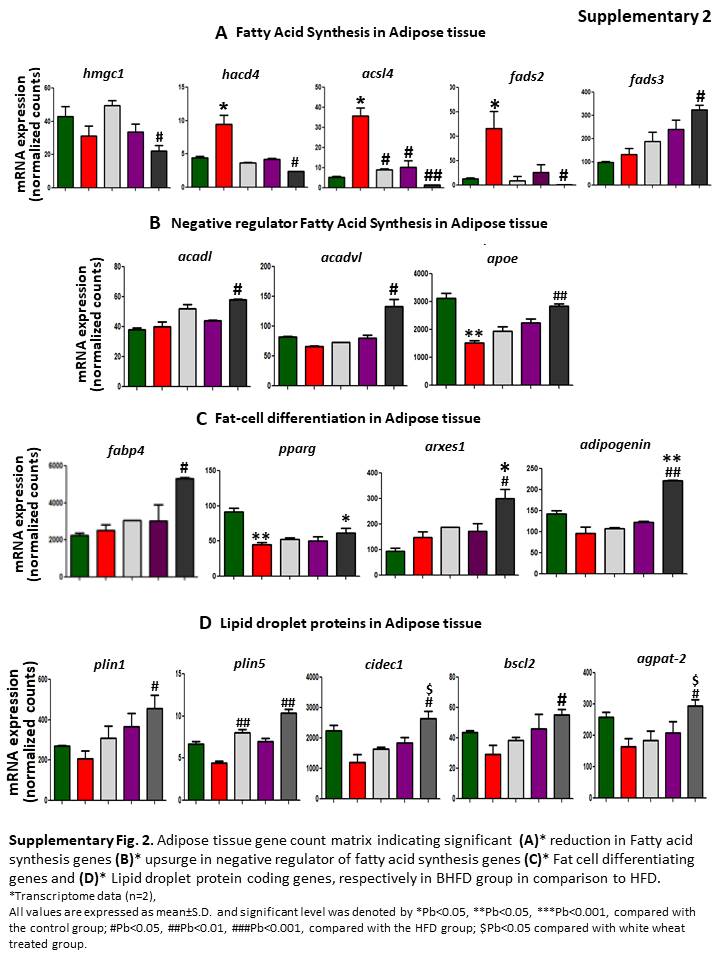

Supplement: Supplementary file 2 — Supporting information [file MNFR-64-1900999-s002.jpg]

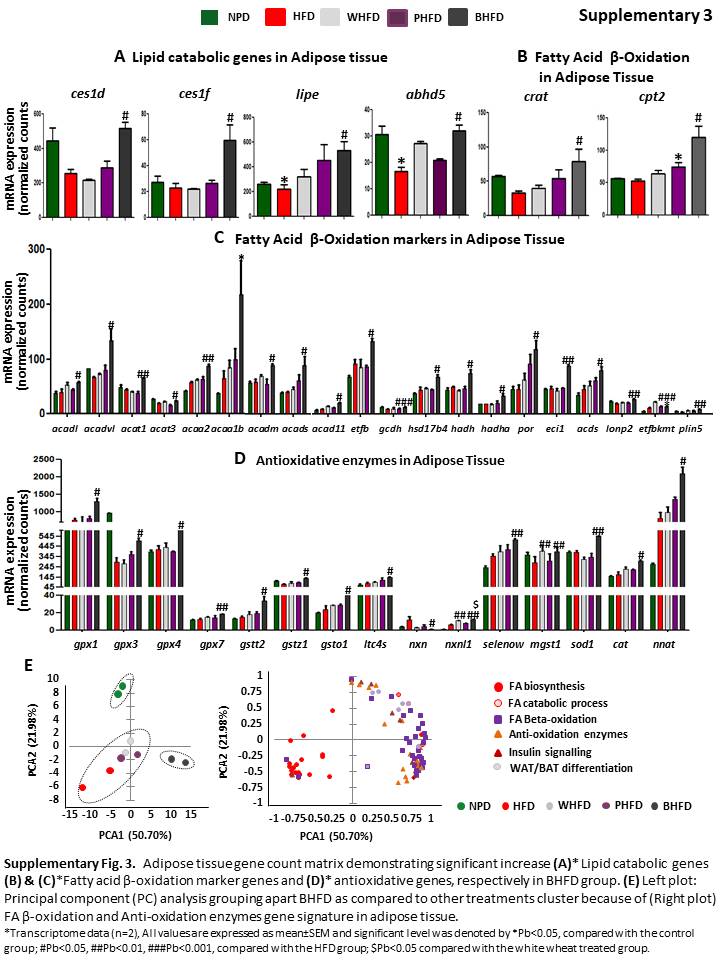

Supplement: Supplementary file 3 — Supporting information [file MNFR-64-1900999-s003.jpg]

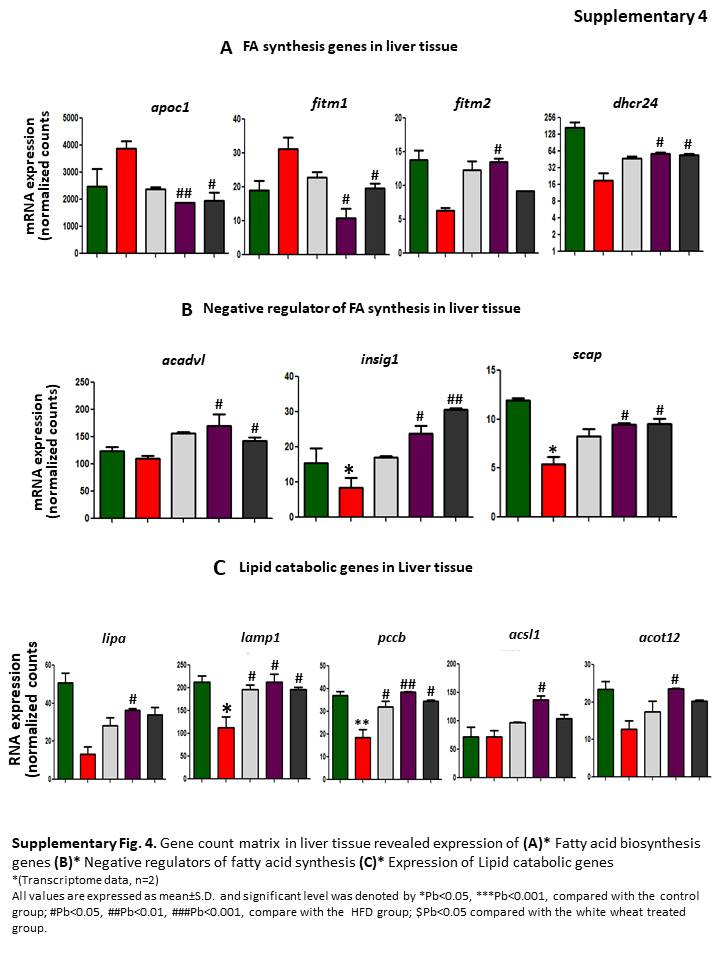

Supplement: Supplementary file 4 — Supporting information [file MNFR-64-1900999-s004.jpg]

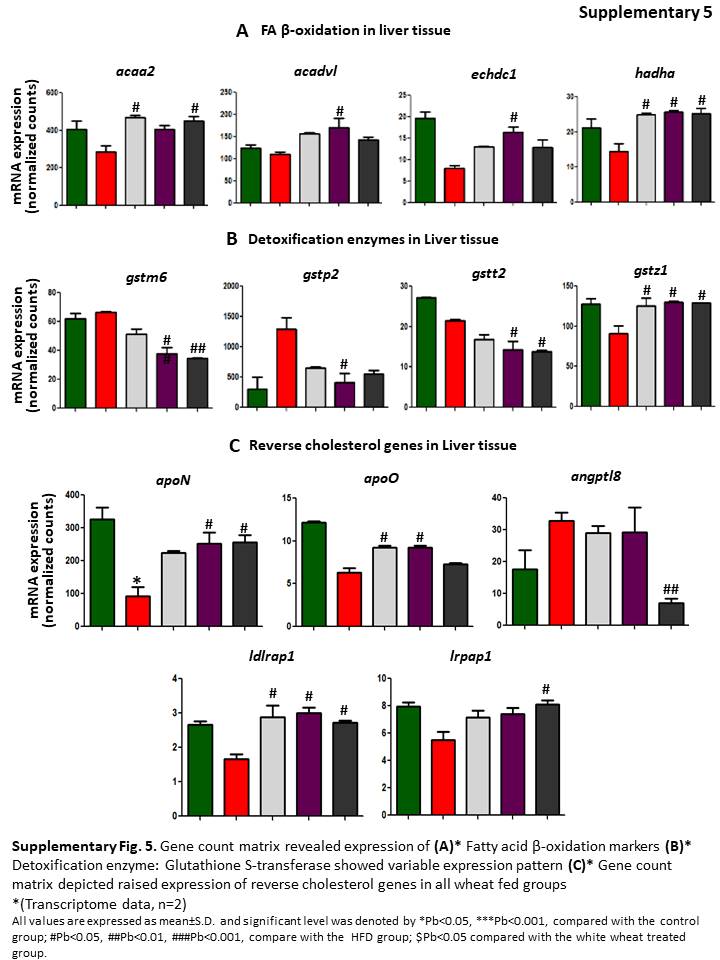

Supplement: Supplementary file 5 — Supporting information [file MNFR-64-1900999-s005.jpg]

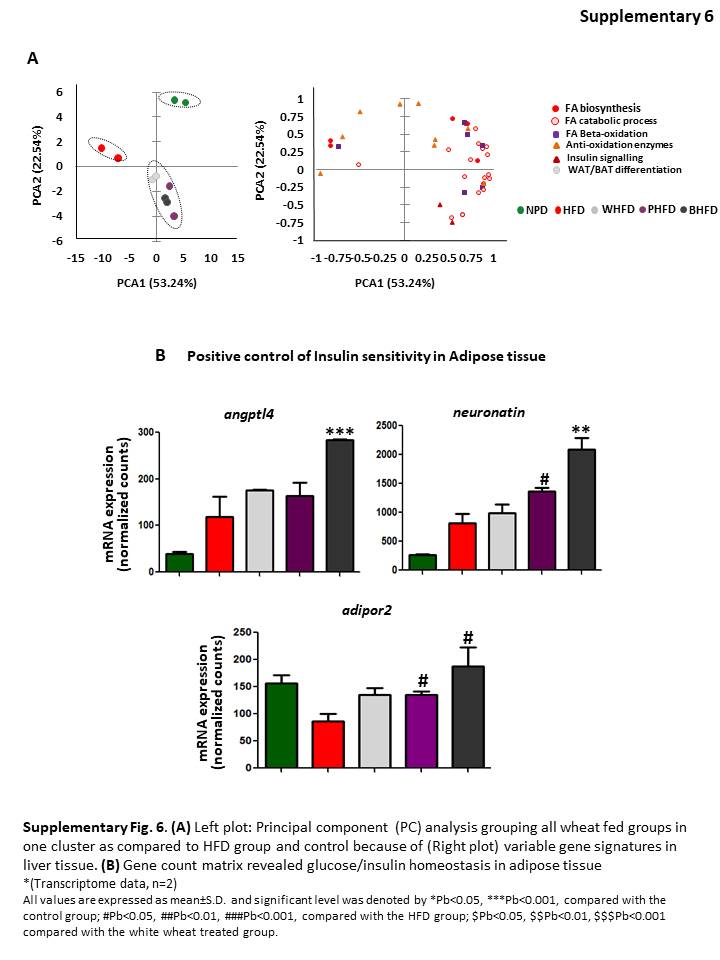

Supplement: Supplementary file 6 — Supporting information [file MNFR-64-1900999-s006.jpg]

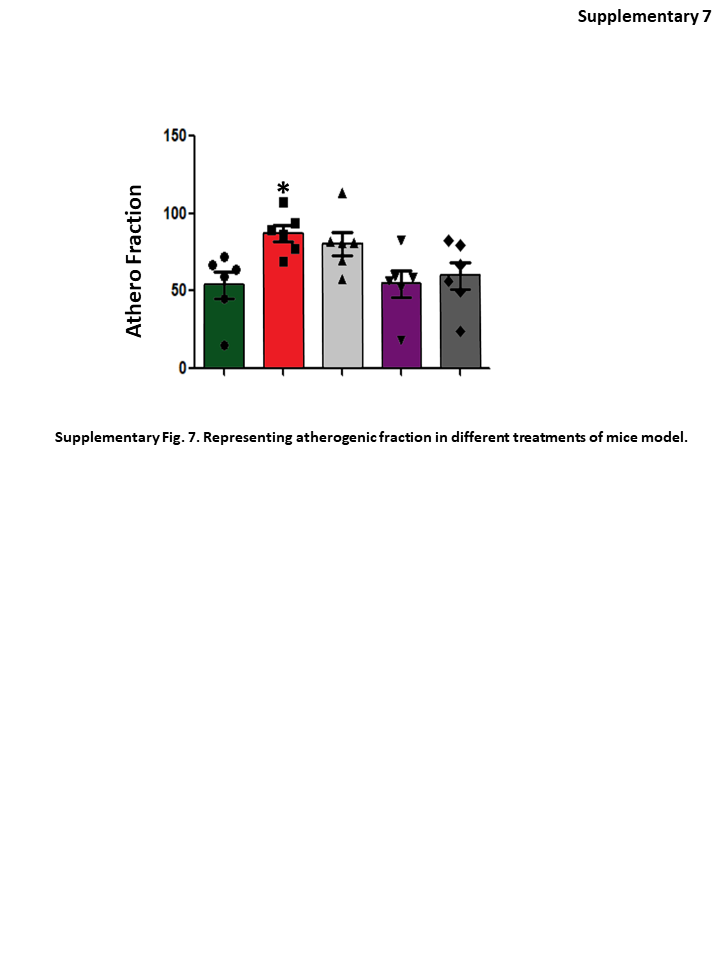

Supplement: Supplementary file 7 — Supporting information [file MNFR-64-1900999-s007.TIF]
