# Supplementary material for: Anthocyanin‐Biofortified Colored Wheat Prevents High Fat Diet–Induced Alterations in Mice: Nutrigenomics Studies
Source: Mol Nutr Food Res. 2020 May 18;64(13):1900999. doi: 10.1002/mnfr.201900999 (PMC7507204; doi:10.1002/mnfr.201900999)
Supplement: Supplementary file 8 — Supporting information [file MNFR-64-1900999-s008.docx]

**Table S1: Diet composition per 100g (For High-fat induced obesity mice-model)**

| **Ingredients**  **(gram)** | **Normal Diet**  **(Control)** | **High-fat diet** | **High-fat diet (with wheat replacement)**  **(WHFD, PHFD and BHFD)** |
| --- | --- | --- | --- |
| ***NPD/Wheat**  (as per diet) | **100** | 36.5 | 30.7 |
| **Lard** | - | 31.0 | 31.33 |
| **^*^Cellulose/Bran**  (as per diet) | - | 2.0 | 3.50 |
| **Casein** | - | 25.0 | 29.14 |
| **Vit/Min Mixture** | - | 4.0 | 4.0 |
| **DL-methionine** | - | 0.30 | 0.30 |
| **Yeast extract** | - | 0.10 | 0.10 |
| **Sodium chloride** | - | 0.10 | 0.10 |
| **Total** | **100g** | **99.0g** | **99.17g** |
| **% Energy and their sources** | | | |
| **Carbohydrate** | 68.90 | 17.42 | 17.43 |
| **Protein** | 23.42 | 25.60 | 25.63 |
| **Fat** | 7.68 | 56.94 | 56.94 |
| **Total** | 100 | 100 | 100 |
| **Kcal/g in diet** | **3.51** | **5.1** | **5.1** |

*In High Fat-diet 36.5% NPD is supplemented,

Whereas in High-fat diet with wheat replacements NPD is replaced with 30.7% of respective

Whole Wheat flours and cellulose is replaced with respective brans.

**Table S2: Energy constituent’s evaluation of by PBTI (NABL accreditation approved)**

| **Parameters** | **Wheat**  **(average)*** | **NPD** | **Method/ Specification/**  **Standard followed** |
| --- | --- | --- | --- |
| **Energy (Kcal/100g)** | 354.5 | 351.48 | PBTI/SOP/18/TP-11 |
| **Carbohydrate %** | 72 | 60.54 |  |
| **Protein %** | 11 | 20.58 | IS 7219 : 1973 |
| **Fat %** | 2.5 | 3 | IS 7874 |

***Average of wheat varities**

**Table S3: Diet composition per 100g (For Oxidative-stress mice-model)**

| **Ingredients**  **(gram)** | **Normal Diet**  **(Control)** | **Wheat replacement -Diet**  **(WW, PW and BW)** |
| --- | --- | --- |
| **NPD/Wheat** (as per diet) | **100** | 84.1 |
| **Cellulose/Bran** | - | 3.6 |
| **Casein** | - | 11.33 |
| **Soybean Oil** | - | 0.9 |
| **Total** | **100g** | **99.93g** |
| **% Energy and their sources** | | |
| **Carbohydrate** | 68.90 | 68.90 |
| **Protein** | 23.42 | 23.42 |
| **Fat** | 7.68 | 7.68 |
| **Total** | **100** | **100** |
| **Kcal/g in diet** | **3.51** | **3.51** |

**Table S4: DEGs in liver and adipose tissue of mouse samples (FDR <0.05; LogFC cutoff 1)**

| **Control vs treatments in Adipose tissue** | | | |
| --- | --- | --- | --- |
| **Treatments** | **Up regulated** | **Down regulated** | **Total** |
| CA vs HFD-A | 1154 | 614 | 1768 |
| CA vs WHFD-A | 49 | 96 | 145 |
| CA vs PHFD-A | 214 | 211 | 425 |
| CA vs BHFD-A | 549 | 640 | 1189 |
| **Control vs treatments in Liver tissue** | | | |
| **Treatments** | **Up regulated** | **Down regulated** | **Total** |
| CL vs HFD-L | 143 | 289 | 432 |
| CL vs WHFD-L | 32 | 73 | 105 |
| CL vs PHFD-L | 37 | 82 | 119 |
| CL vs BHFD-L | 93 | 107 | 200 |

CA-Control adipose tissue, HFD-A = Adipose tissue of treatment group fed with high fat diet, WHFD-A = Adipose tissue of treatment group fed with white wheat and high fat diet, PHFD-A = Adipose tissue of treatment group fed with purple wheat and high fat diet, BHFD-A = Adipose tissue of treatment group fed with black wheat and high fat diet, CL-Control liver tissue, HFD-L = Liver tissue of treatment group fed with high fat diet, WHFD-L = Liver tissue of treatment group fed with white wheat and high fat diet, PHFD-L = Liver tissue of treatment group fed with purple wheat and high fat diet, BHFD-L = Liver tissue of treatment group fed with black wheat and high fat diet

**Table S5: Pathways description-Fig. 1D (Reactome database)**

| **Sr. No.** | **Pathway name** | **Relevant function** |
| --- | --- | --- |
| 1. | **Regulation of cholesterol biosynthesis by SREBP (SREBF)/ Activation of gene expression by SREBF (SREBP)** | Sterol regulatory element binding proteins (SREBPs, SREBFs) respond to low cholesterol concentrations by transiting to the nucleus and activating genes involved in cholesterol and lipid biosynthesis (reviewed in Brown and Goldstein 2009, Osborne and Espenshade 2009, Weber et al. 2004). Newly synthesized SREBPs are transmembrane proteins that bind SCAP in the endoplasmic reticulum (ER) membrane. SCAP binds cholesterol which causes a conformational change that allows SCAP to interact with INSIG, retaining the SCAP: SREBP complex in the ER. INSIG binds oxysterols, which cause INSIG to bind SCAP and retain SCAP:SREBP in the endoplasmic reticulum. In low cholesterol (below about 5 mol%) SCAP no longer interacts with cholesterol or INSIG and binds Sec24 of the CopII coat complex instead. Thus SCAP:SREBP transits with the CopII complex from the ER to the Golgi. In the Golgi SREBP is cleaved by S1P and then by S2P, releasing the N-terminal fragment of SREBP into the cytosol. The N-terminal fragment is imported to the nucleus by importin-beta and then acts with other factors, such as SP1 and NF-Y, to activate transcription of target genes. Targets of SREBP include the genes encoding all enzymes of cholesterol biosynthesis and several genes involved in lipogenesis. SREBP2 most strongly activates cholesterol biosynthesis while SREBP1C most strongly activates lipogenesis |
| 2. | **PPARA activates gene expression/Regulation of lipid metabolism by PPAR-alpha** | The set of genes regulated by PPAR-alpha is not fully known in humans, however many examples have been found in mice. Genes directly activated by PPAR-alpha contain peroxisome proliferator receptor elements (PPREs) in their promoters and include: 1) genes involved in fatty acid oxidation and ketogenesis (Acox1, Cyp4a, Acadm, Hmgcs2);2) genes involved in fatty acid transport (Cd36, , Slc27a1, Fabp1, Cpt1a, Cpt2);3) genes involved in producing fatty acids and very low density lipoproteins (Me1, Scd1);4) genes encoding apolipoproteins (Apoa1, Apoa2, Apoa5);5) genes involved in triglyceride clearance ( Angptl4);6) genes involved in glycerol metabolism (Gpd1 in mouse);7) genes involved in glucose metabolism (Pdk4);8) genes involved in peroxisome proliferation (Pex11a);9) genes involved in lipid storage (Plin, Adfp).Many other genes are known to be regulated by PPAR-alpha but whether their regulation is direct or indirect remains to be found. These genes include: ACACA, FAS, SREBP1, FADS1, DGAT1, ABCA1, PLTP, ABCB4, UGT2B4, SULT2A1, Pnpla2, Acsl1, Slc27a4, many Acot genes, and others (reviewed in Rakhshandehroo et al. 2010). |
| 3. | **BMAL1: Clock, NPAS2 activates circadian gene expression** | As inferred from mouse, BMAL1:CLOCK (ARNTL:CLOCK) and BMAL1:NPAS2 (ARNTL:NPAS2) heterodimers bind to sequence elements (E boxes) in the promoters of target genes and enhance transcription (Gekakis et al. 1998, reviewed in Munoz and Baler 2003). |
| 4. | **RORA activates gene expression** | As inferred from mouse, RORA binds ROR elements (ROREs) in DNA and recruits the coactivators PPARGC1A (PGC-1alpha) and p300 (EP300, a histone acetylase) to activate transcription. |
| 5. | **Fatty acyl-CoA biosynthesis/ Fatty acid metabolism** | -Fatty acyl-CoA biosynthesis involves following steps: -Palmitate synthesis catalyzed by Acetyl-CoA carboxylase and Fatty acid synthase-Conversion of palmitic acid to long chain fatty acids and-Conversion of long chain fatty acids to fatty acyl-CoA by acyl-CoA synthases.  -The synthesis and breakdown of fatty acids are a central part of human energy metabolism, and the eicosanoid class of fatty acid derivatives regulate diverse processes in the body (Vance & Vance 2008 - URL). Processes annotated in this module include the synthesis of fatty acids from acetyl-CoA, mitochondrial and peroxisomal breakdown of fatty acids, and the metabolism of eicosanoids and related molecules |
| 6. | **Acyl chain remodelling of DAG and TAG** | Acyl chain remodeling of triacylglycerol (TAG) and diacylglycerol (DAG) progresses through their hydrolysis by patatin-like phospholipase domain-containing proteins 2/3 (PNPLA2/3). DAG is reacylated back to TAG by acylglycerol O-acyltransferase 1/2 (DGAT1/2), while DAG and its hydrolysis product 2-monoacylglycerol (2-MAG) are transacylated back to TAG by PNPLA2/3. In addition, the DAG hydrolysis product 2-MAG is subsequently hydrolyzed to fatty acid and glycerol by monoglyceride lipase (MGLL) (Jenkins et al. 2004). |
| 7. | **Triglyceride biosynthesis /Triglyceride metabolism** | -The overall process of triglyceride (triacylglycerol) biosynthesis consists of four biochemical pathways: fatty acyl-CoA biosynthesis, conversion of fatty acyl-CoA to phosphatidic acid, conversion of phosphatidic acid to diacylglycerol, and conversion of diacylglycerol to triacylglycerol.  - Fatty acids derived from the diet and synthesized de novo in the liver are assembled into triglycerides (triacylglycerols) for transport and storage. Synthesis proceeds in steps of conversion of fatty acyl-CoA to phosphatidic acid, conversion of phosphatidic acid to diacylglycerol, and conversion of diacylglycerol to triacylglycerol (Takeuchi & Reue 2009).Hydrolysis of triacylglycerol to yield fatty acids and glycerol is a tightly regulated part of energy metabolism. A central part in this regulation is played by hormone-sensitive lipase (HSL), a neutral lipase abundant in adipocytes and skeletal and cardiac muscle, but also abundant in ovarian and adrenal tissue, where it mediates cholesterol ester hydrolysis, yielding cholesterol for steroid biosynthesis. The hormones to which it is sensitive include catecholamines (e.g., epinephrine), ACTH, and glucagon, all of which trigger signaling cascades that lead to its phosphorylation and activation, and insulin, which sets off events leading to its dephosphorylation and inactivation (Kraemer & Shen 2002).The processes of triacylglycerol and cholesterol ester hydrolysis are also regulated by subcellular compartmentalization: these lipids are packaged in cytosolic particles and the enzymes responsible for their hydrolysis, and perhaps for additional steps in their metabolism, are organized at the surfaces of these particles (e.g., Brasaemle et al. 2004). |
| 8. | **Synthesis of very long-chain fatty acyl-CoAs** | Very long-chain fatty acids (VLCFA), ones with more than 20 carbon atoms, have diverse physiological roles, notably as components of ceramides in membrane lipids and as precursors of the eicosanoid hormones that play central roles in the generation and resolution of inflammatory responses. Saturated and monounsaturated VLCFAs can be synthesized by elongation odd palmitic acid synthesized de novo or derived from the diet. Polyunsaturated VLCFAs are synthesized from dietary linoleic and linolenic acids - humans lack the desaturase enzymes to synthesize these molecules from stearate. Chemically, the elongation process that yields VLCFA parallels the one by which palmitate (16 carbons) or stearates (18 carbons) are synthesized de novo from acetate. The starting fatty acid is activated by conjugation with coenzyme A (CoA-SH), condensed with malonyl-CoA to form a 3-oxoacyl CoA containing two more carbon atoms than the starting long chain fatty acyl CoA and CO2, reduced with NADPH to a 3-hydroxyacyl CoA, dehydrated to a trans 2,3-enoyl-CoA, and reduced with NADPH to yield a fatty acyl-CoA two carbons longer than the starting one. The process differs from the de novo one in that the enzymatic activities responsible for each step are expressed by different proteins associated with the endoplasmic reticulum membrane, not by separate domains of a single multifunctional cytosolic protein. In humans, activation is catalysed by one of five acyl-CoA synthetase long-chain (ACSL) enzymes, conjugation by one of seven elongation of very long chain fatty acids (ELOVL) proteins, reduction by one of two HSB17B estradiol dehydrogenases, dehydration by one of four protein tyrosine phosphatase-like / 3-hydroxyacyl-CoA dehydratase (PTPL / HACD) proteins, and reduction by one of two trans-2,3-enoyl-CoA reductase (TECR) proteins. Members of the four enzyme families differ in their tissue-specific expression patterns and in their substrate preferences (chain length, degree of saturation), leading to tissue-specific complements of VLCA (Jakobsson et al. 2006; Kihara 2012; Nugteren 1965).Here the full two-carbon elongation cycle to form stearate from palmitate is annotated, as well as the activation and condensation steps for elongation of arachidonate, the 20-carbon unsaturated fatty acid that plays a central role in the synthesis of prostaglandins and related hormones. |
| 9. | **LDL clearance/Chylomicron clearance/ Plasma lipoprotein clearance** | - LDL (low-density lipoproteins) are complexes of a single molecule of apoprotein B-100 (apoB-100) non-covalently associated with triacylglycerol, free cholesterol, cholesterol esters, and phospholipids. Clearance of LDL from the blood involves binding to LDL receptors associated with coated pits at the cell surface, forming complexes that are internalized and passed via clathrin-coated vesicles to endosomes, where they dissociate. The LDL particles move into lysosomes and are degraded while the LDL receptors are returned to the cell surface. This process occurs in most cell types but is especially prominent in hepatocytes. It plays a major role in returning cholesterol from peripheral tissues to the liver (Hobbs et al. 1990)  -Circulating chylomicrons acquire molecules of apolipoproteins C and E and through interaction with endothelial lipases lose a large fraction of their triacylglycerol. These changes convert them to chylomicron remnants which bind to LDL receptors, primarily on the surfaces of liver cells, clearing them from the circulation (Redgrave 2004).Most very-low-density lipoproteins (VLDL) are converted to low-density lipoproteins (LDL) (VLDL remodeling pathway). Small fractions are taken up by VLDL receptors on extrahepatic cells, as annotated here. Clearance of LDL from the blood involves binding to LDL receptors associated with coated pits at the cell surface, forming complexes that are internalized and passed via clathrin-coated vesicles to endosomes, where they dissociate. The LDL particles move into lysosomes and are degraded while the LDL receptors are returned to the cell surface. This process occurs in most cell types but is especially prominent in hepatocytes. It plays a major role in returning cholesterol from peripheral tissues to the liver (Hobbs et al. 1990).Clearance of circulating HDL particles involves particle binding to cell-surface SR-BI receptors, particle disassembly with rlease of pre-beta HDL (Silver & Tall 2001), and uptake of the latter mediated by cell-surface CUBN:AMN complex (Kozyraki et al. 1999).VLDLR internalization plays a clinically significant role in determining the efficiency of lipoprotein clearance from the blood (Poirier et al. 2008). |
| 10. | **Plasma lipoprotein assembly, remodelling and clearance** | Because of their hydrophobicity, lipids are found in the extracellular spaces of the human body primarily in the form of lipoprotein complexes. Chylomicrons form in the small intestine and transport dietary lipids to other tissues in the body. Very low density lipoproteins (VLDL) form in the liver and transport triacylglycerol synthesized there to other tissues of the body. As they circulate, VLDL are acted on by lipoprotein lipases on the endothelial surfaces of blood vessels, liberating fatty acids and glycerol to be taken up by tissues and converting the VLDL first to intermediate density lipoproteins (IDL) and then to low density lipoproteins (LDL). IDL and LDL are cleared from the circulation via a specific cell surface receptor, found in the body primarily on the surfaces of liver cells. High density lipoprotein (HDL) particles initially formed primarily by the liver, shuttle several kinds of lipids between tissues and other lipoproteins. Notably, they are responsible for the so-called reverse transport of cholesterol from peripheral tissues to LDL for return to the liver. Three aspects of lipoprotein function are currently annotated in Reactome: chylomicron-mediated lipid transport, LDL endocytosis and degradation, and HDL-mediated lipid transport, each divided into assembly, remodeling, and clearance sub pathways. |
| 11. | **Assembly of active LPL and LIPC lipase complexes/ Plasma lipoprotein remodelling** | Lipoprotein lipase (LPL) and hepatic triacylglycerol lipase (LIPC) enzymes on the lumenal surfaces of capillary endothelia mediate the hydrolysis of triglyceride molecules in circulating lipoprotein particles. LPL is widely expressed in the body and is especially abundant in adipocytes and skeletal and cardiac myocytes. Activation of the protein requires glycosylation, dimerization, and glycosylphosphatidylinositol-anchored high density lipoprotein-binding protein 1 (GPIHBP1), which delivers it to heparan sulfate proteoglycan (HSPG) associated with the plasma membrane. It is inactivated by proteolytic cleavage (Berryman & Bensadoun 1995; Sukonina et al. 2006; Young et al. 2011).Expression of the LPL gene is transcriptionally regulated by Cyclic AMP-responsive element-binding protein 3-like protein 3 (CREB3L3), which also regulates the expression of APOA4, APOA5, APOC2, CIDEC and FGF21 (Lee et al. 2011).Maturation of LIPC enzyme requires association with LMF1 protein (or possibly, inferred from sequence similarity, LMF2). Heparin binding stabilizes LIPC in its active dimeric form (Babilonia-Rosa & Neher 2014; Ben-Zeev et al. 2011). |
| 12. | **Transcriptional regulation of white adipocyte differentiation** | Adipogenesis is the process of cell differentiation by which preadipocytes become adipocytes. During this process the preadipocytes cease to proliferate, begin to accumulate lipid droplets and develop morphologic and biochemical characteristics of mature adipocytes such as hormone responsive lipogenenic and lipolytic programs. The most intensively studied model system for adipogenesis is differentiation of the mouse 3T3-L1 preadipocyte cell line by an induction cocktail of containing mitogens (insulin/IGF1), glucocorticoid (dexamethasone), an inducer of cAMP (IBMX), and fetal serum (Cao et al. 1991, reviewed in Farmer 2006). More recently additional cellular models have become available to study adipogenesis that involve almost all stages of development (reviewed in Rosen and MacDougald 2006). In vivo knockout mice lacking putative adipogenic factors have also been extensively studied. Human pathways are traditionally inferred from those discovered in mouse but are now beginning to be validated in cellular models derived from human adipose progenitors (Fischer-Posovszky et al. 2008, Wdziekonski et al. 2011).Adipogenesis is controlled by a cascade of transcription factors (Yeh et al. 1995, reviewed in Farmer 2006, Gesta et al. 2007). One of the first observable events during adipocyte differentiation is a transient increase in expression of the CEBPB (CCAAT/Enhancer Binding Protein Beta, C/EBPB) and CEBPD (C/EBPD) transcription factors (Cao et al. 1991, reviewed in Lane et al. 1999). This occurs prior to the accumulation of lipid droplets. However, it is the subsequent inductions of CEBPA and PPARG that are critical for morphological, biochemical and functional adipocytes.Ectopic expression of CEBPB alone is capable of inducing substantial adipocyte differentiation in fibroblasts while CEBPD has a minimal effect. CEBPB is upregulated in response to intracellular cAMP (possibly via pCREB) and serum mitogens (possibly via Krox20). CEBPD is upregulated in response to glucocorticoids. The exact mechanisms that upregulate the CEBPs are not fully known.CEBPB and CEBPD act directly on the Peroxisome Proliferator-activated Receptor Gamma (PPARG) gene by binding its promoter and activating transcription. CEBPB and CEBPD also directly activate the EBF1 gene (and possibly other EBFs) and KLF5 (Jimenez et al. 2007, Oishi 2005). The EBF1 and KLF5 proteins, in turn bind, and activate the PPARG promoter. Other hormones, such as insulin, affect PPARG expression and other transcription factors, such as ADD1/SREBP1c, bind the PPARG promoter. This is an area of ongoing research.During adipogenesis the PPARG gene is transcribed to yield 2 variants. The adipogenic variant 2 mRNA encodes 30 additional amino acids at the N-terminus compared to the widely expressed variant 1 mRNA.PPARG encodes a type II nuclear hormone receptor (remains in the nucleus in the absence of ligand) that forms a heterodimer with the Retinoid X Receptor Alpha (RXRA). The heterodimer was initially identified as a complex regulating the aP2/FABP4 gene and named ARF6 (Tontonoz et al. 1994).The PPARG:RXRA heterodimer binds a recognition sequence that consists of two hexanucleotide motifs (DR1 motifs) separated by 1 nucleotide. Binding occurs even in the absence of ligands, such as fatty acids, that activate PPARG. In the absence of activating ligands, the PPARG:RXRA complex recruits repressors of transcription such as SMRT/NCoR2, NCoR1, and HDAC3 (Tontonoz and Spiegelman 2008).Each molecule of PPARG can bind 2 molecules of activating ligands. Although, the identity of the endogenous ligands of PPARG is unknown, exogenous activators include fatty acids and the thiazolidinedione class of antidiabetic drugs (reviewed in Berger et al. 2005, Heikkinen et al. 2007, Lemberger et al. 1996). The most potent activators of PPARG in vitro are oxidized derivatives of unsaturated fatty acids.. Upon binding activating ligands PPARG causes a rearrangement of adjacent factors: Corepressors such as SMRT/NCoR2 are lost and coactivators such as TIF2, PRIP, CBP, and p300 are recruited (Tontonoz and Spiegelman). PPARG also binds directly to the TRAP220 subunit of the TRAP/Mediator complex that recruits RNA polymerase II. Thus binding of activating ligand by PPARG causes transcription of PPARG target genes.Targets of PPARG include genes involved in differentiation (PGAR/HFARP, Perilipin, aP2/FABP4, CEBPA), fatty acid transport (LPL, FAT/CD36), carbohydrate metabolism (PEPCK-C, AQP7, GK, GLUT4 (SLC2A4)), and energy homeostasis (LEPTIN and ADIPONECTIN) (Perera et al. 2006).Within 10 days of differentiation CEBPB and CEBPD are no longer located at the PPARG promoter. Instead CEBPA is present. EBF1 and PPARG bind the CEBPA promoter and activate transcription of CEBPA, one of the key transcription factors in adipogenesis. A current hypothesis posits a self-reinforcing loop that maintains PPARG expression and the differentiated state: PPARG activates CEBPA and CEBPA activates PPARG. Additionally EBF1 (and possibly other EBFs) activates CEBPA, CEBPA activates EBF1, and EBF1 activates PPARG. |
| 13. | **ChREBP activates metabolic gene expression** | ChREBP (Carbohydrate Response Element Binding Protein) is a large multidomain protein containing a nuclear localization signal near its amino terminus, polyproline domains, a basic helix-loop-helix-leucine zipper domain, and a leucine-zipper-like domain (Uyeda et al., 2002). Its dephosphorylation in response to molecular signals associated with the well-fed state allows it to enter the nucleus, interact with MLX protein, and bind to ChRE DNA sequence motifs near Acetyl-CoA carboxylase, Fatty acid synthase, and Pyruvate kinase (L isoform) genes (Ishi et al.2004). |

**Table S6. Pathways description-Fig. 2A (Reactome database)**

| **Sr. No.** | **Pathway name** | **Relevant function** |
| --- | --- | --- |
| 14. | **Transcriptional activation of mitochondrial biogenesis** | PGC-1α (peroxisome-proliferator-activated receptor γ co-activator-1α) is a co-transcriptional regulation factor that induces mitochondrial biogenesis by activating different transcription factors, including nuclear respiratory factor 1 and nuclear respiratory factor 2, which activate mitochondrial transcription factor A. Phosphorylated PPARGC1A (PGC-1alpha) does not bind DNA directly but instead interacts with other transcription factors, notably NRF1 and NRF2 (via HCF1). NRF1 and NRF2 together with PPARGC1A activate the transcription of nuclear-encoded, mitochondrially targeted proteins such as TFB2M, TFB1M, and TFAM. PGC-1beta and PPRC appear to act similarly to PGC-1alpha but have not been as well studied. Transcription of PPARGC1A itself is upregulated by CREB1 (in response to calcium), MEF2C/D, ATF2, and PPARGC1A. |
| 15. | **Metallothioneins bind metals** | Metallothioneins are highly conserved, cysteine-rich proteins that bind metals via thiolate bonds (recent general reviews in Capdevila et al. 2012, Blindauer et al. 2014, reviews of mammalian metallothioneins in Miles et al. 2000, Maret 2011, Vasak and Meloni 2011, Thirumoorthy et al. 2001, Babula et al. 2012). Mammals contain 4 general metallothionein isoforms (MT1,2,3,4). The MT1 isoform has radiated in primates to 8 or 9 functional proteins (depending on classification of MT1L). Each mammalian metallothionein binds a total of 7 divalent metal ions in two clusters, the alpha and beta clusters. Though the functions of metallothioneins have not been fully elucidated, they appear to participate in detoxifying heavy metals (reviewed in Sharma et al. 2013), storing and transporting zinc, and redox biochemistry. Metallothioneins interact with many other cellular proteins, with most interactions involving proteins of the central nervous system (reviewed in Atrian and Capdevila 2013). |
| 16. | **FMO oxidises nucleophiles** | Flavin-containing monooxygenases (FMOs) are the second family of microsomal oxidative enzymes with broad and overlapping specificity. The major reactions FMOs catalyze are nucleophilic hetero-atom compounds such as nitrogen, sulfur or phosphorus as the hetero-atom to form N-oxides, S-oxides or P-oxides respectively. Despite the functional overlap with cytochrome P450s, the mechanism of action differs. FMOs bind and activate molecular oxygen before the substrate binds to the enzyme (picture). They also require flavin adenosine dinucleotide (FAD) as a cofactor. Unlike cytochrome P450 enzymes, FMOs are heat-labile, a useful way to distinguish which enzyme system is at work for researchers studying metabolism. Also, FMOs are not inducible by substrates, unlike the P450 enzymes.\n(1) NADPH binds to the enzyme and reduces the prosthetic group FAD to FADH2. NADP+ remains bound to the enzyme.\n(2) Incorporation of molecular oxygen to form a hydroperoxide.\n(3) A peroxide oxygen is transferred to the substrate.\n(4) Water is released.\n(5) NADP+ dissociates returning the enzyme to its initial state.\n\nTo date, there are 6 isozymes of FMO (FMO1-6) in humans, the most prominent and active one being FMO3. The FMO6 gene does not encode for a functional enzyme although it has the greatest sequence similarity with FMO3 (71%), whilst the others range from 50-58% sequence similarity with FMO3. FMO1-3 are the ones that exhibit activity towards nucleophiles, the others are insignificant in this respect (Cashman 2003, Krueger & Williams 2005). |
| 17. | **NR1D1 (Rev-ERBA) represses gene expression** | REV-ERBA binds DNA elements very similar to those bound by the transcription activator RORA. RORAREV-ERBA bound to DNA and heme recruits the corepressors NCoR and HDAC3 to repress transcription. Thus REV-ERBA and RORA appear to compete to repress or activate genes, repectively. |
| 18. | **Biological oxidations** | All organisms are constantly exposed to foreign chemicals every day. These can be man-made (drugs, industrial chemicals) or natural (alkaloids, toxins from plants and animals). Uptake is usually via ingestion but inhalation and transdermal routes are also common.The very nature of many chemicals that make them suitable for uptake by these routes, in other words their lipophilicty (favours fat solubility) is also the main reason organisms have developed mechanisms that convert them to hydrophilic (favours water solubility) compounds which are readily excreted via bile and urine. Otherwise, lipophilic chemicals would accumulate in the body and overwhelm defense mechanisms. This process is called biotransformation and is catalyzed by enzymes mainly in the liver of higher organisms but a number of other organs have considerable ability to process xenobiotica such as kidneys, gut and lungs. As well as xenobiotics, many endogenous compounds are commonly eliminated by this process.This mechanism is of ancient origin and a major factor for its development in animals is plants. Most animals are plant eaters and thus are subject to a huge variety of chemical compounds which plants produce to stop themselves being eaten. This complex set of enzymes have several features which make them ideal for biotransformation;(1) metabolites of the parent chemical are usually made more water soluble so it favours rapid excretion via bile and urine(2) the enzymes possess broad and overlapping specificity to be able to deal with newly exposed chemicals(3) metabolites of the parent generally don't have adverse biological effects. In the real world however, all these criteria have exceptions. Many chemicals are transformed into reactive metabolites. In pharmacology, the metabolites of some parent drugs exert the desired pharmacological effect but in the case of polycyclic aromatic hydrocarbons (PAHs), which can undergo epoxidation, it results in the formation of an electrophile which can attack proteins and DNA. Metabolism of xenobiotica occurs in several steps called Phase 1 (functionalization) and Phase 2 (conjugation). To improve water solubility, a functional group is added to or exposed on the chemical in one or more steps (Phase 1) to which hydrophilic conjugating species can be added (Phase 2). Functional groups can either be electrophilic (epoxides, carbonyl groups) or nucleophilic (hydroxyls, amino and sulfhydryl groups, carboxylic groups) (see picture).Once chemicals undergo functionalization; the electrophilic or nucleophilic species can be detrimental to biological systems. Electrophiles can react with electron-rich macromolecules such as proteins, DNA and RNA by covalent interaction whilst nucleophiles have the potential to interact with biological receptors. That's why conjugation is so important as it mops up these potentially reactive species. Many chemicals, when exposed to certain metabolizing enzymes can induce those enzymes, a process called enzyme induction. The effect of this is that these chemicals accelerate their own biotransformation and excretion. The reverse is also true where some chemicals cause enzyme inhibition. Some other factors that alter enzyme levels are sex, age and genetic predisposition. Between species, there can be considerable differences in biotransformation ability which is a problem faced by drug researchers interpreting toxicological results to humans. |

**Table S7. Pathways description-Fig. 4A (Reactome database)**

| **Sr. No.** | **Pathway name** | **Relevant function** |
| --- | --- | --- |
| 19. | **Activation of gene expression SREBF (SREBP)/ Regulation of cholesterol biosynthesis by SREBP (SREBF)** | After transiting to the nucleus SREBPs (SREBP1A/1C/2, SREBFs) bind short sequences, sterol regulatory elements (SREs), in the promoters of target genes (reviewed in Eberle et al. 2004, Weber et al. 2004). SREBPs alone are relatively weak activators of transcription, with SREBP1C being significantly weaker than SREBP1A or SREBP2. In combination with other transcription factors such as SP1 and NF-Y the SREBPs are much stronger activators. SREBP1C seems to more specifically target genes involved in fatty acid synthesis while SREBP2 seems to target genes involved in cholesterol synthesis (Pai et al. 1998). |
| 20. | **Cholesterol biosynthesis via desmosterol/lathosterol** | The transformation of zymosterol into cholesterol can follow either of routes, one in which reduction of the double bond in the isooctyl side chain is the final step (cholesterol synthesis via desmosterol, also known as the Bloch pathway) and one in which this reduction is the first step (cholesterol biosynthesis via lathosterol, also known as the Kandutsch-Russell pathway). The former pathway is prominent in the liver and many other tissues while the latter is prominent in skin, where it may serve as the source of the 7-dehydrocholesterol that is the starting point for the synthesis of D vitamins (Mitsche et al. 2015). |
| 21. | **Cholesterol biosynthesis** | Cholesterol is synthesized de novo from acetyl CoA. The overall synthetic process is outlined in the attached illustration. Enzymes whose regulation plays a major role in determining the rate of cholesterol synthesis in the body are highlighted in red, and connections to other metabolic processes are indicated. The transformation of zymosterol into cholesterol can follow either of routes, one in which reduction of the double bond in the isooctyl side chain is the final step (cholesterol synthesis via desmosterol, also known as the Bloch pathway) and one in which this reduction is the first step ( via lathosterol, also known as the Kandutsch-Russell pathway). The former pathway is prominent in the liver and many other tissues while the latter is prominent in skin, where it may serve as the source of the 7-dehydrocholesterol that is the starting point for the synthesis of D vitamins. Defects in several of the enzymes involved in this process are associated with human disease and have provided useful insights into the regulatory roles of cholesterol and its synthetic intermediates in human development (Gaylor 2002; Herman 2003; Kandutsch & Russell 1960; Mitsche et al. 2015; Song et al. 2005). |
| 22. | **Endogenous sterols** | A number of CYPs take part in cholesterol biosynthesis and elimination, thus playing an important role in maintaining cholesterol homeostasis. Under normal physiological conditions, cholesterol intake (diet or synthesized de novo from acetyl CoA) equals cholesterol elimination (degraded to bile salts, secreted in bile and used in steroid hormone synthesis). These processes are under tight regulatory control and any disruption leads to increased cholesterol levels resulting in cardiovacular disease. The CYPs involved in cholesterol homeostasis could serve as potential targets for cholesterol-lowering drugs (Lewis 2004, Guengerich 2006, Pikuleva 2006). |
| 23. | **Metabolism of steroids** | Steroids, defined by a four-ring cyclopenta[a]phenanthrene carbon skeleton, include cholesterol and bile acids and salts, steroid hormones, and vitamin D, three groups of molecules synthesized from it. In this module, pathways for the synthesis of cholesterol from HMG-CoA (hydroxymethylglutaryl-coenzyme A) (Russell 1992), and for its conversion to bile acids and salts (Russell 2003), steroid hormones (Payne & Hales 2004), and vitamin D (Dusso et al. 2005) are annotated, together with the SREBP-mediated regulatory process that normally links the rate of cholesterol synthesis to levels of cellular cholesterol (Brown & Goldstein 2009). |
| 24. | **PPARA activates gene expression/Regulation of lipid metabolism by PPARA** | The set of genes regulated by PPAR-alpha is not fully known in humans, however many examples have been found in mice. Genes directly activated by PPAR-alpha contain peroxisome proliferator receptor elements (PPREs) in their promoters and include: 1) genes involved in fatty acid oxidation and ketogenesis (Acox1, Cyp4a, Acadm, Hmgcs2);2) genes involved in fatty acid transport (Cd36, , Slc27a1, Fabp1, Cpt1a, Cpt2);3) genes involved in producing fatty acids and very low density lipoproteins (Me1, Scd1);4) genes encoding apolipoproteins (Apoa1, Apoa2, Apoa5);5) genes involved in triglyceride clearance ( Angptl4);6) genes involved in glycerol metabolism (Gpd1 in mouse);7) genes involved in glucose metabolism (Pdk4);8) genes involved in peroxisome proliferation (Pex11a);9) genes involved in lipid storage (Plin, Adfp).Many other genes are known to be regulated by PPAR-alpha but whether their regulation is direct or indirect remains to be found. These genes include: ACACA, FAS, SREBP1, FADS1, DGAT1, ABCA1, PLTP, ABCB4, UGT2B4, SULT2A1, Pnpla2, Acsl1, Slc27a4, many Acot genes, and others (reviewed in Rakhshandehroo et al. 2010). |
| 25. | **VLDR internalisation and degradation/LDL clearance/Plasma lipoprotein assembly, remodeling and clearance** | The steps involved in proprotein convertase PCSK9-induced degradation of VLDLR are described here (Poirier et al. 2008). The rate of this catabolic process plays a clinically significant role in determining the efficiency of lipoprotein clearance from the blood.  -LDL (low-density lipoproteins) are complexes of a single molecule of apoprotein B-100 (apoB-100) non-covalently associated with triacylglycerol, free cholesterol, cholesterol esters, and phospholipids. Clearance of LDL from the blood involves binding to LDL receptors associated with coated pits at the cell surface, forming complexes that are internalized and passed via clathrin-coated vesicles to endosomes, where they dissociate. The LDL particles move into lysosomes and are degraded while the LDL receptors are returned to the cell surface. This process occurs in most cell types but is especially prominent in hepatocytes. It plays a major role in returning cholesterol from peripheral tissues to the liver (Hobbs et al. 1990).  -Because of their hydrophobicity, lipids are found in the extracellular spaces of the human body primarily in the form of lipoprotein complexes. Chylomicrons form in the small intestine and transport dietary lipids to other tissues in the body. Very low density lipoproteins (VLDL) form in the liver and transport triacylglycerol synthesized there to other tissues of the body. As they circulate, VLDL are acted on by lipoprotein lipases on the endothelial surfaces of blood vessels, liberating fatty acids and glycerol to be taken up by tissues and converting the VLDL first to intermediate density lipoproteins (IDL) and then to low density lipoproteins (LDL). IDL and LDL are cleared from the circulation via a specific cell surface receptor, found in the body primarily on the surfaces of liver cells. High density lipoprotein (HDL) particles initially formed primarily by the liver, shuttle several kinds of lipids between tissues and other lipoproteins. Notably, they are responsible for the so-called reverse transport of cholesterol from peripheral tissues to LDL for return to the liver. Three aspects of lipoprotein function are currently annotated in Reactome: chylomicron-mediated lipid transport, LDL endocytosis and degradation, and HDL-mediated lipid transport, each divided into assembly, remodeling, and clearance subpathways. |
| 26. | **Metabolism of lipids** | Lipids are hydrophobic but otherwise chemically diverse molecules that play a wide variety of roles in human biology. They include ketone bodies, fatty acids, triacylglycerols, phospholipids and sphingolipids, eicosanoids, cholesterol, bile salts, steroid hormones, and fat-soluble vitamins. They function as a major source of energy (fatty acids, triacylglycerols, and ketone bodies), are major constituents of cell membranes (cholesterol and phospholipids), play a major role in their own digestion and uptake (bile salts), and participate in numerous signaling and regulatory processes (steroid hormones, eicosanoids, phosphatidylinositols, and sphingolipids) (Vance & Vance 2008 - URL).The central steroid in human biology is cholesterol, obtained from animal fats consumed in the diet or synthesized de novo from acetyl-coenzyme A. (Vegetable fats contain various sterols but no cholesterol.) Cholesterol is an essential constituent of lipid bilayer membranes and is the starting point for the biosyntheses of bile acids and salts, steroid hormones, and vitamin D. Bile acids and salts are mostly synthesized in the liver. They are released into the intestine and function as detergents to solubilize dietary fats. Steroid hormones are mostly synthesized in the adrenal gland and gonads. They regulate energy metabolism and stress responses (glucocorticoids), salt balance (mineralocorticoids), and sexual development and function (androgens and estrogens). At the same time, chronically elevated cholesterol levels in the body are associated with the formation of atherosclerotic lesions and hence increased risk of heart attacks and strokes. The human body lacks a mechanism for degrading excess cholesterol, although an appreciable amount is lost daily in the form of bile salts and acids that escape recycling. Aspects of lipid metabolism currently annotated in Reactome include lipid digestion, mobilization, and transport; fatty acid, triacylglycerol, and ketone body metabolism; peroxisomal lipid metabolism; phospholipid and sphingolipid metabolism; cholesterol biosynthesis; bile acid and bile salt metabolism; and steroid hormone biosynthesis. |
| 27. | **ABC transporters in lipid homeostasis** | A defined subset of the ABC transporter superfamily, the ABCA transporters, are highly expressed in monocytes and macrophages and are regulated by cholesterol flux which may indicate their role in in chronic inflammatory diseases (Schmitz and Kaminski 2001, Schmitz et al. 2000). Some D and G members of the ABC transporter family are also important in lipid transport (Voloshyna & Reiss 2011, Morita & Imanaka 2012, Morita et al. 2011). |
| 28. | **Transport of small molecules** | The transport of inorganic ions and small water-soluble organic molecules across the lipid bilayer is achieved by specialized transmembrane proteins, each of which is responsible for the transfer of a specific ion, [molecule](https://www.ncbi.nlm.nih.gov/books/n/mboc4/A4754/def-item/A5486/), or group of closely related ions or molecules. (Alberts B et al. 2002) |
| 29. | **Miscellaneous transport and binding events** | This section contains known transport and binding events that as of yet cannot be placed in existing pathways (Purves 2001, He et al. 2009, Rees et al. 2009). |
| 30. | **Transcriptional activation of mitochondrial biogenesis** | PGC-1α (peroxisome-proliferator-activated receptor γ co-activator-1α) is a co-transcriptional regulation factor that induces mitochondrial biogenesis by activating different transcription factors, including nuclear respiratory factor 1 and nuclear respiratory factor 2, which activate mitochondrial transcription factor A. Phosphorylated PPARGC1A (PGC-1alpha) does not bind DNA directly but instead interacts with other transcription factors, notably NRF1 and NRF2 (via HCF1). NRF1 and NRF2 together with PPARGC1A activate the transcription of nuclear-encoded, mitochondrially targeted proteins such as TFB2M, TFB1M, and TFAM. PGC-1beta and PPRC appear to act similarly to PGC-1alpha but have not been as well studied. Transcription of PPARGC1A itself is upregulated by CREB1 (in response to calcium), MEF2C/D, ATF2, and PPARGC1A. Transcription of PPARGC1A is repressed by NR1D1 (REV-ERBA). |
| 31. | **Import of palmitoyl-CoA into the mitochondrial matrix** | The mitochondrial carnitine system catalyzes the transport of long-chain fatty acids into the mitochondrial matrix where they undergo beta oxidation. This transport system consists of the malonyl-CoA sensitive carnitine palmitoyltransferase I (CPT-I) localized in the mitochondrial outer membrane, the carnitine:acylcarnitine translocase, an integral inner membrane protein, and carnitine palmitoyltransferase II localized on the matrix side of the inner membrane. (Kerner and Hoppel, 2000). |
| 32. | **Detoxification of Reactive Oxygen Species** | Reactive oxygen species such as superoxide (O2.-), peroxides (ROOR), singlet oxygen, peroxynitrite (ONOO-), and hydroxyl radical (OH.) are generated by cellular processes such as respiration (reviewed in Murphy 2009, Brand 2010) and redox enzymes and are required for signalling yet they are damaging due to their high reactivity (reviewed in Imlay 2008, Buettner 2011, Kavdia 2011, Birben et al. 2012, Ray et al. 2012). Aerobic cells have defences that detoxify reactive oxygen species by converting them to less reactive products. Superoxide dismutases convert superoxide to hydrogen peroxide and oxygen (reviewed in Fukai and Ushio-Fukai 2011). Catalase and peroxidases then convert hydrogen peroxide to water. Humans contain 3 superoxide dismutases: SOD1 is located in the cytosol and mitochondrial intermembrane space, SOD2 is located in the mitochondrial matrix, and SOD3 is located in the extracellular region. Superoxide, a negative ion, is unable to easily cross membranes and tends to remain in the compartment where it was produced. Hydrogen peroxide, one of the products of superoxide dismutase, is able to diffuse across membranes and pass through aquaporin channels. In most cells the primary source of hydrogen peroxide is mitochondria and, once in the cytosol, hydrogen peroxide serves as a signalling molecule to regulate redox-sensitive proteins such as transcription factors, kinases, phosphatases, ion channels, and others (reviewed in Veal and Day 2011, Ray et al. 2012). Hydrogen peroxide is decomposed to water by catalase, decomposed to water plus oxidized thioredoxin by peroxiredoxins, and decomposed to water plus oxidized glutathione by glutathione peroxidases (Presnell et al. 2013). |
| 33. | **ROS, RNS production in phagocytes** | The first line of defense against infectious agents involves an active recruitment of phagocytes to the site of infection. Recruited cells include polymorhonuclear (PMN) leukocytes (i.e., neutrophils) and monocytes/macrophages, which function together as innate immunity sentinels (Underhill DM & Ozinsky A 2002; Stuart LM & Ezekowitz RA 2005; Flannagan RS et al. 2012). Dendritic cells are also present, serving as important players in antigen presentation for ensuing adaptive responses (Savina A & Amigorena S 2007). These cell types are able to bind and engulf invading microbes into a membrane-enclosed vacuole - the phagosome, in a process termed phagocytosis. Phagocytosis can be defined as the receptor-mediated engulfment of particles greater than 0.5 micron in diameter. It is initiated by the cross-linking of host cell membrane receptors following engagement with their cognate ligands on the target surface (Underhill DM & Ozinsky A 2002; Stuart LM & Ezekowitz RA 2005; Flannagan RS et al. 2012). When engulfed by phagocytes, microorganisms are exposed to a number of host defense microbicidal events within the resulting phagosome. These include the production of reactive oxygen and nitrogen species (ROS and RNS, RONS) by specialized enzymes (Fang FC et al. 2004; Kohchi C et al. 2009; Gostner JM et al. 2013; Vatansever F et al. 2013). NADPH oxidase (NOX) complex consume oxygen to produce superoxide radical anion (O2.-) and hydrogen peroxide (H2O2) (Robinson et al. 2004). Induced NO synthase (iNOS) is involved in the production of NO, which is the primary source of all RNS in biological systems (Evans TG et al. 1996). The NADPH phagocyte oxidase and iNOS are expressed in both PMN and mononuclear phagocytes and both cell types have the capacity for phagosomal burst activity. However, the magnitude of ROS generation in neutrophils far exceeds that observed in macrophages (VanderVen BC et al. 2009). Macrophages are thought to produce considerably more RNS than neutrophils (Fang FC et al. 2004; Nathan & Shiloh 2000).The presence of RONS characterized by a relatively low reactivity, such as H2O2, O2?? or NO, has no deleterious effect on biological environment (Attia SM 2010; Weidinger A & and Kozlov AV 2015) Their activity is controlled by endogenous antioxidants (both enzymatic and non-enzymatic) that are induced by oxidative stress. However the relatively low reactive species can initiate a cascade of reactions to generate more damaging ?secondary? species such as hydroxyl radical (?OH), singlet oxygen or peroxinitrite (Robinson JM 2008; Fang FC et al. 2004). These "secondary" RONS are extremely toxic causing irreversible damage to all classes of biomolecules (Weidinger A & and Kozlov AV 2015; Fang FC et al. 2004; Kohchi C et al. 2009; Gostner JM et al. 2013; Vatansever F et al. 2013).Although macrophages and neutrophils use similar mechanisms for the internalization of targets, there are differences in how they perform phagocytosis and in the final outcome of the process (Tapper H & Grinstein S 1997; Vierira OV et al. 2002). Once formed, the phagosome undergoes an extensive maturation process whereby it develops into a microbicidal organelle able to eliminate the invading pathogen. Maturation involves re-modelling both the membrane of the phagosome and its luminal contents (Vierira OV et al. 2002). In macrophages, phagosome formation and maturation follows a series of strictly coordinated membrane fission/fusion events between the phagosome and compartments of the endo/lysosomal network gradually transforming the nascent phagosome into a phagolysosome, a degradative organelle endowed with potent microbicidal properties (Zimmerli S et al. 1996; Vierira OV et al. 2002). Neutrophils instead contain a large number of preformed granules such as azurophilic and specific granules that can rapidly fuse with phagosomes delivering antimicrobial substances (Karlsson A & Dahlgren C 2002; Naucler C et al. 2002; Nordenfelt P and Tapper H 2011). Phagosomal pH dynamics may also contribute to the maturation process by regulating membrane traffic events. The microbicidal activity of macrophages is characterized by progressive acidification of the lumen (down to pH 4?5) by the proton pumping vATPase. A low pH is a prerequisite for optimal enzymatic activity of most late endosomal/lysosomal hydrolases reported in macrophages. Neutrophil phagosome pH regulation differs significantly from what is observed in macrophages (Nordenfelt P and Tapper H 2011; Winterbourn CC et al. 2016). The massive activation of the oxidative burst is thought to result in early alkalization of neutrophil phagosomes which is linked to proton consumption during the generation of hydrogen peroxide (Segal AW et al. 1981; Levine AP et al. 2015). Other studies showed that neutrophil phagosome maintained neutral pH values before the pH gradually decreased (Jankowski A et al. 2002). Neutrophil phagosomes also exhibited a high proton leak, which was initiated upon activation of the NADPH oxidase, and this activation counteracted phagosomal acidification (Jankowski A et al. 2002).The Reactome module describes ROS and RNS production by phagocytic cells. The module includes cell-type specific events, for example, myeloperoxidase (MPO)-mediated production of hypochlorous acid in neutrophils. It also highlights differences between phagosomal pH dynamics in neutrophils and macrophages. The module describes microbicidal activity of selective RONS such as hydroxyl radical or peroxynitrite however the mechanisms by which reactive oxygen/nitrogen species kill pathogens is still a matter of debate. |

**STable 8. Pathways description-Fig. 5C (Reactome data)**

| **Sr. No.** | **Pathway name** | | **Relevant function** | | |
| --- | --- | --- | --- | --- | --- |
| **Adipose Tissue** | | | | | |
| 34. | **P15P, PP2A AND IER3 Regulate P13K/AKT Signalling** | | Phosphatidylinositol-5-phosphate (PI5P) may modulate PI3K/AKT signaling in several ways. PI5P is used as a substrate for production of phosphatidylinositol-4,5-bisphosphate, PI(4,5)P2 (Rameh et al. 1997, Clarke et al. 2008, Clarke et al. 2010, Clarke and Irvine 2013, Clarke et al. 2015), which serves as a substrate for activated PI3K, resulting in the production of PIP3 (Mandelker et al. 2009, Burke et al. 2011). The majority of PI(4,5)P2 in the cell, however, is produced from the phosphatidylinositol-4-phosphate (PI4P) substrate (Zhang et al. 1997, Di Paolo et al. 2002, Oude Weernink et al. 2004, Halstead et al. 2006, Oude Weernink et al. 2007). PIP3 is necessary for the activating phosphorylation of AKT. AKT1 can be deactivated by the protein phosphatase 2A (PP2A) complex that contains a regulatory subunit B56-beta (PPP2R5B) or B56-gamma (PPP2R5C). PI5P inhibits AKT1 dephosphorylation by PP2A through an unknown mechanism (Ramel et al. 2009). Increased PI5P levels correlate with inhibitory phosphorylation(s) of the PP2A complex. MAPK1 (ERK2) and MAPK3 (ERK1) are involved in inhibitory phosphorylation of PP2A, in a process that involves IER3 (IEX-1) (Letourneux et al. 2006, Rocher et al. 2007). It is uncertain, however, whether PI5P is in any way involved in ERK-mediated phosphorylation of PP2A or if it regulates another PP2A kinase. | | |
| 35. | **IRS-related events triffered by IGF1R** | | The phosphorylated type 1 insulin-like growth factor receptor phosphorylates IR1, IRS2, IRS4 and possibly other IRS/DOK family members (reviewed in Pavelic et al. 2007, Chitnis et al. 2008, Maki et al. 2010, Parrella et al. 2010, Siddle et al. 2012). The phosphorylated IRS proteins serve as scaffolds that bind the effector molecules PI3K and GRB2:SOS. PI3K then activates PKB (AKT) signaling while GRB2:SOS activates RAS-RAF-MAPK signaling. | | |
| 36. | **IRS mediated signalling/P13K Cascade/Signalling by Insulin receptor** | | Release of phospho-IRS from the insulin receptor triggers a cascade of signalling events via PI3K, SOS, RAF and the MAP kinases.  -The PI3K (Phosphatidlyinositol-3-kinase) - AKT signaling pathway stimulates cell growth and survival.  -Insulin binding to its receptor results in receptor autophosphorylation on tyrosine residues and the tyrosine phosphorylation of insulin receptor substrates (e.g. IRS and Shc) by the insulin receptor tyrosine kinase. This allows association of IRSs with downstream effectors such as PI-3K via its Src homology 2 (SH2) domains leading to end point events such as Glut4 (Slc2a4) translocation. Shc when tyrosine phosphorylated associates with Grb2 and can thus activate the Ras/MAPK pathway independent of the IRSs. Signal transduction by the insulin receptor is not limited to its activation at the cell surface. The activated ligand-receptor complex initially at the cell surface, is internalised into endosomes itself a process which is dependent on tyrosine autophosphorylation. Endocytosis of activated receptors has the dual effect of concentrating receptors within endosomes and allows the insulin receptor tyrosine kinase to phosphorylate substrates that are spatially distinct from those accessible at the plasma membrane. Acidification of the endosomal lumen, due to the presence of proton pumps, results in dissociation of insulin from its receptor. (The endosome constitutes the major site of insulin degradation). This loss of the ligand-receptor complex attenuates any further insulin-driven receptor re-phosphorylation events and leads to receptor dephosphorylation by extra-lumenal endosomally-associated protein tyrosine phosphatases (PTPs). The identity of these PTPs is not clearly established yet. | | |
| 37. | **Regulation of Insulin-like Growth Factor (IGF) transport and uptake by IGFBPs** | | The family of Insulin like Growth Factor Binding Proteins (IGFBPs) share 50% amino acid identity with conserved N terminal and C terminal regions responsible for binding Insulin like Growth Factors I and II (IGF I and IGF II). Most circulating IGFs are in complexes with IGFBPs, which are believed to increase the residence of IGFs in the body, modulate availability of IGFs to target receptors for IGFs, reduce insulin like effects of IGFs, and act as signaling molecules independently of IGFs. About 75% of circulating IGFs are in 1500 220 KDa complexes with IGFBP3 and ALS. Such complexes are too large to pass the endothelial barrier. The remaining 20 25% of IGFs are bound to other IGFBPs in 40 50 KDa complexes. IGFs are released from IGF:IGFBP complexes by proteolysis of the IGFBP. IGFs become active after release, however IGFs may also have activity when still bound to some IGFBPs. IGFBP1 is enriched in amniotic fluid and is produced in the liver under control of insulin (insulin suppresses production). IGFBP1 binding stimulates IGF function. It is unknown which if any protease degrades IGFBP1. IGFBP2 is enriched in cerebrospinal fluid; its binding inhibits IGF function. IGFBP2 is not significantly degraded in circulation. IGFB3, which binds most IGF in the body is enriched in follicular fluid and found in many other tissues. IGFBP 3 may be cleaved by plasmin, thrombin, Prostate specific Antigen (PSA, KLK3), Matrix Metalloprotease-1 (MMP1), and Matrix Metalloprotease-2 (MMP2). IGFBP3 also binds extracellular matrix and binding lowers its affinity for IGFs. IGFBP3 binding stimulates the effects of IGFs. IGFBP4 acts to inhibit IGF function and is cleaved by Pregnancy associated Plasma Protein A (PAPPA) to release IGF. IGFBP5 is enriched in bone matrix; its binding stimulates IGF function. IGFBP5 is cleaved by Pregnancy Associated Plasma Protein A2 (PAPPA2), ADAM9, complement C1s from smooth muscle, and thrombin. Only the cleavage site for PAPPA2 is known. IGFBP6 is enriched in cerebrospinal fluid. It is unknown which if any protease degrades IGFBP6. | | |
| **Liver Tissue** | | | | | |
| 38. | **Signalling by Insulin receptor** | | Insulin binding to its receptor results in receptor autophosphorylation on tyrosine residues and the tyrosine phosphorylation of insulin receptor substrates (e.g. IRS and Shc) by the insulin receptor tyrosine kinase. This allows association of IRSs with downstream effectors such as PI-3K via its Src homology 2 (SH2) domains leading to end point events such as Glut4 (Slc2a4) translocation. Shc when tyrosine phosphorylated associates with Grb2 and can thus activate the Ras/MAPK pathway independent of the IRSs.Signal transduction by the insulin receptor is not limited to its activation at the cell surface. The activated ligand-receptor complex initially at the cell surface, is internalised into endosomes itself a process which is dependent on tyrosine autophosphorylation. Endocytosis of activated receptors has the dual effect of concentrating receptors within endosomes and allows the insulin receptor tyrosine kinase to phosphorylate substrates that are spatially distinct from those accessible at the plasma membrane. Acidification of the endosomal lumen, due to the presence of proton pumps, results in dissociation of insulin from its receptor. (The endosome constitutes the major site of insulin degradation). This loss of the ligand-receptor complex attenuates any further insulin-driven receptor re-phosphorylation events and leads to receptor dephosphorylation by extra-lumenal endosomally-associated protein tyrosine phosphatases (PTPs). The identity of these PTPs is not clearly established yet. | | |
| 39. | **Insulin receptor recycling** | | Triggered by acidification of the endosome, insulin dissociates from the receptor and is degraded. The receptor is dephosphorylated and re-integrated into the plasma membrane, ready to be activated again by the binding of insulin molecules. | | |
| 40. | **Regulation of Insulin-like Growth Factor (IGF) transport and uptake by IGFBPs** | | The family of Insulin like Growth Factor Binding Proteins (IGFBPs) share 50% amino acid identity with conserved N terminal and C terminal regions responsible for binding Insulin like Growth Factors I and II (IGF I and IGF II). Most circulating IGFs are in complexes with IGFBPs, which are believed to increase the residence of IGFs in the body, modulate availability of IGFs to target receptors for IGFs, reduce insulin like effects of IGFs, and act as signalling molecules independently of IGFs. About 75% of circulating IGFs are in 1500 220 KDa complexes with IGFBP3 and ALS. Such complexes are too large to pass the endothelial barrier. The remaining 20 25% of IGFs are bound to other IGFBPs in 40 50 KDa complexes. IGFs are released from IGF:IGFBP complexes by proteolysis of the IGFBP. IGFs become active after release, however IGFs may also have activity when still bound to some IGFBPs. IGFBP1 is enriched in amniotic fluid and is produced in the liver under control of insulin (insulin suppresses production). IGFBP1 binding stimulates IGF function. It is unknown which if any protease degrades IGFBP1. IGFBP2 is enriched in cerebrospinal fluid; its binding inhibits IGF function. IGFBP2 is not significantly degraded in circulation. IGFB3, which binds most IGF in the body is enriched in follicular fluid and found in many other tissues. IGFBP 3 may be cleaved by plasmin, thrombin, Prostate specific Antigen (PSA, KLK3), Matrix Metalloprotease-1 (MMP1), and Matrix Metalloprotease-2 (MMP2). IGFBP3 also binds extracellular matrix and binding lowers its affinity for IGFs. IGFBP3 binding stimulates the effects of IGFs. IGFBP4 acts to inhibit IGF function and is cleaved by Pregnancy associated Plasma Protein A (PAPPA) to release IGF. IGFBP5 is enriched in bone matrix; its binding stimulates IGF function. IGFBP5 is cleaved by Pregnancy Associated Plasma Protein A2 (PAPPA2), ADAM9, complement C1s from smooth muscle, and thrombin. Only the cleavage site for PAPPA2 is known. IGFBP6 is enriched in cerebrospinal fluid. It is unknown which if any protease degrades IGFBP6. | | |
| **STable 9: Genes description along with function in Adipose tissue** | | | | | |
| **Gene** | | **Full name** | | | **Function** |
| Hmgcs1 | | 3-hydroxy-3-methylglutaryl-Coenzyme A synthase 1 | | | [cholesterol biosynthetic process](http://www.informatics.jax.org/vocab/gene_ontology/GO:0006695) |
| Hmgcs2 | | 3-hydroxy-3-methylglutaryl-Coenzyme A synthase 2 | | |  |
| Hacd4 | | 3-hydroxyacyl-CoA dehydratase 4 | | | [Fatty acid elongation](http://www.informatics.jax.org/vocab/gene_ontology/GO:0030497) ([very long-chain fatty acid biosynthetic process](http://www.informatics.jax.org/vocab/gene_ontology/GO:0042761)) |
| Acsl4 | | acyl-CoA synthetase long-chain family member 4 | | | FA biosynthesis process |
| Fads2 | | fatty acid desaturase 2 | | | FA biosynthesis process |
| Fads3 | | fatty acid desaturase 3 | | | FA biosynthesis process |
| Acsf3 | | Acyl-CoA synthetase family member3 | | | FA biosynthesis process  Catalyzes the initial reaction in intramitochondrial fatty acid synthesis, by activating malonate and methylmalonate, but not acetate, into their respective CoA thioester. May have some preference toward very-long-chain substrates. |
| Acadl | | acyl-coenzyme dehydrogenase, long chain | | | Negative regulation of fatty acid biosynthetic process |
| Acadvl | | acyl-coenzyme dehydrogenase, very long chain | | | Negative regulation of fatty acid biosynthetic process |
| Apoe | | Apolipoprotein E | | | [Negative regulation of cholesterol biosynthetic process](http://www.informatics.jax.org/vocab/gene_ontology/GO:0045541)  it is highly expressed in adipocytes and has positive relation with body fat mass  its deficiency shows impaired lipoproteins internalization and triglyceride accumulation |
| [Fabp4](http://www.informatics.jax.org/marker/MGI:88038) | | fatty acid binding protein 4, adipocyte  (AP2) | | | [Brown fat cell differentiation](http://www.informatics.jax.org/vocab/gene_ontology/GO:0050873)  Homozygotes for a targeted null mutation exhibit susceptibility to diet-induced obesity, attenuated dibutyryl cAMP-induced adipocyte release of glycerol and free fatty acid, and reduced acute insulin secretion in response to beta-adrenergic stimulation |
| Pparg | | Peroxisome proliferator activated receptor gamma | | | [Positive regulation of fat cell differentiation and fatty acid oxidation](http://www.informatics.jax.org/vocab/gene_ontology/GO:0046321) |
| Arxes1 | | adipocyte-related X-chromosome expressed sequence 1 | | | Results in differentiation of preadipocyte |
| [Adig](http://www.informatics.jax.org/marker/MGI:2675492) | | adipogenin | | | [Positive regulation of fat cell differentiation](http://www.informatics.jax.org/vocab/gene_ontology/GO:0045600) |
| Plp1 | | Perilipin 1 | | | Perilipin encodes lipid droplet surface protein |
| Plp5 | | Perilipin 5 | | |  |
| Cidec | | cell death-inducing DFFA-like effector c | | | Lipid droplet organization |
| Bscl 2 | | Berardinelli-Seip congenital lipodystrophy 2 (seipin) | | | [Positive regulation of cold-induced thermogenesis](http://www.informatics.jax.org/vocab/gene_ontology/GO:0120162)  Mice homozygous for a knock-out allele exhibit severe generalized lipodystrophy with hepatic steatosis, glucose intolerance, and insulin resistance |
| Agpat 2 | | 1-acylglycerol-3-phosphate O-acyltransferase 2 (lysophosphatidic acid acyltransferase, beta) | | | Lipid metabolic process  Mice homozygous for a knock-out allele exhibit loss of white and brown adipose tissue, insulin resistance, and hepatic steatosis. |
| Acadl | | acyl-coenzyme dehydrogenase, long chain | | | [Fatty acid beta-oxidation using acyl-CoA dehydrogenase](http://www.informatics.jax.org/vocab/gene_ontology/GO:0033539) |
| Acadvl | | acyl-coenzyme dehydrogenase, very long chain | | | [Fatty acid beta-oxidation using acyl-CoA dehydrogenase](http://www.informatics.jax.org/vocab/gene_ontology/GO:0033539) |
| Acat1 | | acetyl-Coenzyme A acetyltransferase 1 | | | Fatty acid beta-oxidation |
| Acat2 | | acetyl-Coenzyme A acetyltransferase 2 | | | Fatty acid beta-oxidation |
| Acat3 | | acetyl-Coenzyme A acetyltransferase 3 | | | Fatty acid beta-oxidation |
| Acaa2 | | acetyl-Coenzyme A acetyltransferase 2  (mitochondrial 3-oxoacyl-Coenzyme A thiolase) | | | Fatty acid beta-oxidation |
| Acaa1b | | acetyl-Coenzyme A acyltransferase 1B | | | Fatty acid beta-oxidation |
| Acadm | | acyl-coenzyme dehydrogenase, medium chain | | | [Fatty acid beta-oxidation using acyl-CoA dehydrogenase](http://www.informatics.jax.org/vocab/gene_ontology/GO:0033539) |
| Acads | | acyl-Coenzyme A dehydrogenase, short chain | | | [Fatty acid beta-oxidation using acyl-CoA dehydrogenase](http://www.informatics.jax.org/vocab/gene_ontology/GO:0033539) |
| Acad11 | | acyl-Coenzyme A dehydrogenase, 11 subunit | | | [Fatty acid beta-oxidation using acyl-CoA dehydrogenase](http://www.informatics.jax.org/vocab/gene_ontology/GO:0033539) |
| [Etfb](http://www.informatics.jax.org/marker/MGI:106098) | | electron transferring flavoprotein, beta polypeptide | | | [Fatty acid beta-oxidation using acyl-CoA dehydrogenase](http://www.informatics.jax.org/vocab/gene_ontology/GO:0033539) |
| Gcdh | | glutaryl-Coenzyme A dehydrogenase | | | [Fatty acid beta-oxidation using acyl-CoA dehydrogenase](http://www.informatics.jax.org/vocab/gene_ontology/GO:0033539) |
| Hsd17b4 | | hydroxysteroid (17-beta) dehydrogenase 4 | | | Fatty acid beta-oxidation |
| Hadh | | hydroxyacyl-Coenzyme A dehydrogenase | | | Fatty acid beta-oxidation |
| Hadha | | Hydroxyacyl-Coenzyme A dehydrogenase/3-ketoacyl-Coenzyme A thiolase/enoyl-Coenzyme A hydratase (trifunctional protein), alpha sununit | | | Fatty acid beta-oxidation |
| Por | | P450 (cytochrome) oxidoreductase | | | Fatty acid oxidation |
| Eci1 | | Enoyl-Coenzyme A delta isomerase 1 | | | Fatty acid beta-oxidation |
| Acbd4 | | acyl-Coenzyme A binding domain containing 4 | | | Peroxisomal lipid metabolism |
| Lonp2 | | lon peptidase 2, peroxisomal | | | Regulation of Fatty acid beta-oxidation  In human, peroxisomes function primarily to catalyze fatty acid beta-oxidation and, as a by-product, produce hydrogen peroxide and superoxide. The protein encoded by this gene is an ATP-dependent protease that likely plays a role in maintaining overall peroxisome homeostasis as well as proteolytically degrading peroxisomal proteins damaged by oxidation. |
| [Etfbkmt](http://www.informatics.jax.org/marker/MGI:2443575) | | electron transfer flavoprotein beta subunit lysine methyltransferase | | | [Negative regulation of fatty acid beta-oxidation using acyl-CoA dehydrogenase](http://www.informatics.jax.org/vocab/gene_ontology/GO:1904736) |
| Plin5 | | perilipin 5 | | | Negative regulation of triglyceride catabolic process and  Positive regulation of fatty acid oxidation |
| Cpt2 | | carnitine palmitoyltransferase 2 | | | Obligate enzymes required for long chain mitochondrial fatty acid β-oxidation |
| Crat | | carnitine acetyltransferase | | | Obligate enzymes required for long chain mitochondrial fatty acid β-oxidation  Mice with muscle specific loss of function display increased circulating glucose level, impaired glucose tolerance, insulin resistance, decreased circulating triglyceride and free fatty acid levels, increased susceptibility to diet-induced obesity and abnormal mitochondrial physiology |
| Gpx1 | | glutathione peroxidase 1 | | | [cell redox homeostasis](http://www.informatics.jax.org/vocab/gene_ontology/GO:0045454), intrinsic apoptotic signalling pathway in response to oxidative stress |
| Gpx3 | | glutathione peroxidase 3 | | | response to oxidative stress |
| [Gpx4](http://www.informatics.jax.org/marker/MGI:104767) | | glutathione peroxidase 4 | | | response to oxidative stress |
| [Gpx7](http://www.informatics.jax.org/marker/MGI:104767) | | glutathione peroxidase 7 | | | response to oxidative stress |
| Glutathione S-transferases (GSTs; [EC 2.5.1.18](http://enzyme.expasy.org/EC/2.5.1.18)) are a family of enzymes that play an important role in detoxification by catalyzing the conjugation of many hydrophobic and electrophilic compounds with reduced glutathione. Based on their biochemical, immunologic, and structural properties, the mammalian cytosolic GSTs are divided into several classes, including alpha (e.g., [138359](https://www.omim.org/entry/138359)), **mu** (e.g., [138350](https://www.omim.org/entry/138350)), kappa ([602321](https://www.omim.org/entry/602321)), **theta** (e.g., [600436](https://www.omim.org/entry/600436)), **pi**, omega (e.g., [605482](https://www.omim.org/entry/605482)), and **zeta** (e.g., [603758](https://www.omim.org/entry/603758)). In addition, there is a class of microsomal GSTs (e.g., [138330](https://www.omim.org/entry/138330)). Each class is encoded by a single gene or a gene family. | | | | | |
| Gstt2 | | glutathione S-transferase, theta 2 | | |  |
| Gstz1 | | glutathione transferase zeta 1 (maleylacetoacetate isomerase) | | |  |
| Gsto1 | | glutathione S-transferase, omega1 | | |  |
| [Mgst1](http://www.informatics.jax.org/marker/MGI:1913850) | | microsomal glutathione S-transferase 1 | | | [cellular response to lipid hydroperoxide](http://www.informatics.jax.org/vocab/gene_ontology/GO:0071449) |
| Ltc4s | | leukotriene C4 synthase | | | mice homozygous for disruption in this gene display abnormal inflammatory and hypersensitivity reactions but are otherwise normal |
| Nxn | | nucleoredoxin | | | [cell redox homeostasis](http://www.informatics.jax.org/vocab/gene_ontology/GO:0045454)  Nucleoredoxin guards against oxidative stress by protecting antioxidant enzyme. |
| Nxnl1 | | nucleoredoxin-like 1 | | |  |
| [Selenow](http://www.informatics.jax.org/marker/MGI:95994) | | selenoprotein w | | | [cell redox homeostasis](http://www.informatics.jax.org/vocab/gene_ontology/GO:0045454) |
| Sod | | superoxide dismutase 1, soluble | | | positive regulation of oxidative stress-induced intrinsic apoptotic signalling pathway, removal of superoxide radicals |
| Cat | | catalase | | | In adipose tissue of obese mice, the expression of catalase, an anti-oxidant enzyme, significantly decreases, which may cause insufficient elimination of hydrogen peroxide, but it does not in liver or skeletal muscle |
| Nnat | | neuronatin | | | It’s a proteolipid. Antinfflamatory, [positive regulation of insulin secretion](http://www.informatics.jax.org/vocab/gene_ontology/GO:0032024) |
| Ces1d | | carboxylesterase 1D | | | Lipid catabolic process, [short-chain fatty acid catabolic process](http://www.informatics.jax.org/vocab/gene_ontology/GO:0019626) |
| Ces1f | | carboxylesterase 1f | | | Lipid catabolic process, [short-chain fatty acid catabolic process](http://www.informatics.jax.org/vocab/gene_ontology/GO:0019626) |
| [Lipe](http://www.informatics.jax.org/marker/MGI:96790) | | lipase, hormone sensitive | | | [long-chain fatty acid catabolic process](http://www.informatics.jax.org/vocab/gene_ontology/GO:0042758) |
| Abhd5 | | abhydrolase domain containing 5 | | | Positive regulator of triglyceride catabolic process and lipoprotein lipase activity  [fatty acid metabolic process](http://www.informatics.jax.org/vocab/gene_ontology/GO:0006631) |
| Angptl4 | | angiopoietin-like 4 | | | The encoded protein is induced by peroxisome proliferation activators and functions as a serum hormone that regulates glucose homeostasis, lipid metabolism, and insulin sensitivity. Decreased expression of this gene has been associated with type 2 diabetes. |
| Adipor2 | | adiponectin receptor 2 | | | Maintain fat and [glucose homeostasis](http://www.informatics.jax.org/vocab/gene_ontology/GO:0042593) |
| **STable 10: Genes description along with function in Liver tissue** | | | | | |
| **Gene** | | **Full name** | | **function** | |
| ApoCI | | apolipoprotein CI | | Positive regulator of fatty acid biosynthetic process  It was found to be a potent inhibitor of CETP. (Plasma cholesteryl ester transfer protein -CETP) | |
| Fitm1 | | fat storage inducing transmembrane protein 1 | | Phospholipid biosynthesis  Plays important role in lipid droplet accumulation | |
| Fitm2 | | fat storage inducing transmembrane protein 2 | | Regulation of triglyceride biosynthetic process | |
| Dhcr24 | | 24-dehydrocholesterol reductase | | Cholesterol metabolic process | |
| Acadvl | | acyl-coenzyme dehydrogenase, very long chain | | Positive regulation of fatty acid biosynthetic process | |
| Insig2 | | Insulin induced gene 2 | | Positive regulation of fatty acid biosynthetic process | |
| Scap | | SREBF chaperon | | Regulate fatty acid biosynthetic process and essential for sterol synthesis  HDAC3 and SCAP control symbiotic pathways of liver lipid metabolism that are critical for suppression of lipotoxicity. | |
| Lipa | | Liposomal/Lysosomal acid lipase A | | Lipid catabolic process | |
| Lamp1 | | lysosomal-associated membrane protein 1 | | Lipid catabolic process | |
| Pccb | | propionyl Coenzyme A carboxylase, beta polypeptide | | Lipid catabolic process | |
| Acsl1 | | acyl-CoA synthetase long-chain family member 1 | | [Long-chain fatty acid catabolic process](http://www.informatics.jax.org/vocab/gene_ontology/GO:0042758), [triglyceride biosynthetic process](http://www.informatics.jax.org/vocab/gene_ontology/GO:0019432), Liver acyl-CoA levels are reduced when this gene is conditionally knocked out in the liver. | |
| Acot2 | | acyl-CoA thioesterase | | Very long-chain fatty acid catabolic process | |
| Acaa2 | | acetyl-Coenzyme A acyltranferase 2 (mitochondrial 3-oxoacyl-Coenzyme A thiolase) | | Fatty acid beta-oxidation | |
| Acadvl | | acyl-coenzyme dehydrogenase, very long chain | | Fatty acid beta-oxidation | |
| Echdca1 | | enoyl Coenzyme A hydratase domain containing 1 | | Fatty acid beta-oxidation | |
| Hadha | | hydroxyacyl-Coenzyme A dehydrogenase/3-ketoacyl-Coenzyme A thiolase/enoyl-Coenzyme A hydratase (trifunctional protein), alpha subunit | | Fatty acid beta-oxidation | |
| Glutathione S-transferases (GSTs; [EC 2.5.1.18](http://enzyme.expasy.org/EC/2.5.1.18)) are a family of enzymes that play an important role in detoxification by catalyzing the conjugation of many hydrophobic and electrophilic compounds with reduced glutathione. Based on their biochemical, immunologic, and structural properties, the mammalian cytosolic GSTs are divided into several classes, including alpha (e.g., [138359](https://www.omim.org/entry/138359)), mu (e.g., [138350](https://www.omim.org/entry/138350)), kappa ([602321](https://www.omim.org/entry/602321)), theta (e.g., [600436](https://www.omim.org/entry/600436)), pi, omega (e.g., [605482](https://www.omim.org/entry/605482)), and zeta (e.g., [603758](https://www.omim.org/entry/603758)). In addition, there is a class of microsomal GSTs (e.g., [138330](https://www.omim.org/entry/138330)). Each class is encoded by a single gene or a gene family. | | | | | |
| Gstm2 | | glutathione S-transferase, mu 2 | | The mu class of glutathione S-transferase functions in the detoxification of electrophilic compounds, including carcinogens, therapeutic drugs, environmental toxins and products of oxidative stress, by conjugation with glutathione. | |
| Gstm6 | | glutathione S-transferase, mu 6 | |  |  |
| Gstp2 | | glutathione S-transferase, Pi 2 | |  |  |
| Gstt2 | | glutathione S-transferase, theta 2 | |  |  |
| **Apolipoproteins** are multifunctional proteins that serve as templates for the assembly of lipoprotein particles, maintain their structure and direct their metabolism through binding to membrane receptors and regulation of enzyme activity. The three principal functions of lipoproteins are contribution to interorgan fuel (triglyceride) distribution (by means of the fuel transport pathway), to the maintenance of the extracellular cholesterol pool (by means of the overflow pathway) and reverse cholesterol transport ( [Dominiczak](https://www.ncbi.nlm.nih.gov/pubmed/?term=Dominiczak%20MH%5BAuthor%5D&cauthor=true&cauthor_uid=22028427) et al., 2011) | | | | | |
| ApoO | | Apolipoprotein O | | The encoded protein associates with HDL, LDL and VLDL lipoproteins and is characterized by chondroitin-sulfate glycosylation. This protein may be involved in preventing lipid accumulation in the myocardium in obese and diabetic patients. | |
| ApoN | | Apolipoprotein N  Also known as D10Ucla2 | | Extracellular protein | |
| Angptl8 | | Angiopoietin-like protein 8 | | Hormone that acts as a blood lipid regulator by regulating serum triglyceride levels  Knockout experiments in mouse also support a role of ANGPTL8 in regulating serum triglyceride levels without affecting glucose homeostasis | |
| Ldlrap1 | | low density lipoprotein receptor adaptor protein 1 | | Homozygous mutant mice have increased levels of circulating LDL cholesterol and total plasma cholesterol and are physiologically similar to humans with autosomal recessive hypercholesterolemia (ARH) | |
